# The wheat *Sr22*, *Sr33*, *Sr35* and *Sr45* genes confer resistance against stem rust in barley

**DOI:** 10.1111/pbi.13460

**Published:** 2020-09-06

**Authors:** M. Asyraf Md Hatta, Sanu Arora, Sreya Ghosh, Oadi Matny, Mark A. Smedley, Guotai Yu, Soma Chakraborty, Dhara Bhatt, Xiaodi Xia, Burkhard Steuernagel, Terese Richardson, Rohit Mago, Evans S. Lagudah, Nicola J. Patron, Michael Ayliffe, Matthew N. Rouse, Wendy A. Harwood, Sambasivam Periyannan, Brian J. Steffenson, Brande B.H. Wulff

**Affiliations:** ^1^ John Innes Centre Norwich Research Park Norwich UK; ^2^ Department of Agriculture Technology Faculty of Agriculture Universiti Putra Malaysia Serdang Malaysia; ^3^ Department of Plant Pathology Stakman Borlaug Center for Sustainable Plant Health University of Minnesota St. Paul MN USA; ^4^ Commonwealth Scientific and Industrial Research Organization (CSIRO) Agriculture and Food Canberra ACT Australia; ^5^ The Earlham Institute Norwich Research Park Norwich UK; ^6^ USDA‐ARS Cereal Disease Laboratory St. Paul MN USA

**Keywords:** Ug99, stem rust, wheat, barley, *Sr22*, *Sr33*, *Sr35*, *Sr45*, stacking, durable disease resistance

## Abstract

In the last 20 years, stem rust caused by the fungus *Puccinia graminis* f. sp. *tritici* (*Pgt*), has re‐emerged as a major threat to wheat and barley production in Africa and Europe. In contrast to wheat with 60 designated stem rust (*Sr*) resistance genes, barley’s genetic variation for stem rust resistance is very narrow with only ten resistance genes genetically identified. Of these, only one complex locus consisting of three genes is effective against TTKSK, a widely virulent *Pgt* race of the Ug99 tribe which emerged in Uganda in 1999 and has since spread to much of East Africa and parts of the Middle East. The objective of this study was to assess the functionality, in barley, of cloned wheat *Sr* genes effective against race TTKSK. *Sr22*, *Sr33*, *Sr35* and *Sr45* were transformed into barley cv. Golden Promise using *Agrobacterium*‐mediated transformation. All four genes were found to confer effective stem rust resistance. The barley transgenics remained susceptible to the barley leaf rust pathogen *Puccinia hordei*, indicating that the resistance conferred by these wheat *Sr* genes was specific for *Pgt*. Furthermore, these transgenic plants did not display significant adverse agronomic effects in the absence of disease. Cloned *Sr* genes from wheat are therefore a potential source of resistance against wheat stem rust in barley.

## Introduction

Wheat stem rust, caused by the fungus *P*.* graminis* f. sp. *tritici* (*Pgt*), is one of the major threats to barley (*Hordeum vulgare*) production in North America (Steffenson, 1992) and Australia (Dill‐Macky *et al*., 1991). This destructive fungal disease can cause a significant reduction in plant growth and yield of both barley and wheat (De Wolf *et al*., 2011). In 1999, a new virulent isolate of *Pgt* called Ug99 (typed as race TTKSK according to Jin *et al*., 2008) was detected in Uganda which had overcome *Sr31*, a widely deployed stem rust resistance gene in bread wheat (*Triticum aestivum*; Pretorius *et al*., 2000). At that time Ug99 and its derivatives were virulent on more than 80% of the world’s wheat cultivars (Singh *et al*., 2008). In recent years new *Pgt* races, that are not members of the Ug99 race group, have caused disease outbreaks on wheat in Europe (including Germany; Olivera Firpo *et al*., 2017, and Italy; Bhattacharya, 2017), Asia (Russia; Shamanin *et al*., 2016) and Africa (Ethiopia; Olivera *et al*., 2015).

Effective ways of controlling this disease include fungicide application and breeding for resistant cultivars (McIntosh *et al*., 1995), with this latter strategy being the most cost‐effective and environmentally acceptable. However, when lines carrying a single resistance (*R*) gene effective against a specific disease are deployed, strong selection pressure is imposed on the pathogen population often leading to the development of mutants capable of overcoming the resistance and the outbreak of an epidemic (Stakman, 1957). Notwithstanding, there are a few cases where *R* genes effective against *Pgt* have shown remarkable durability despite being deployed as a single gene for many years over a wide area where the pathogen is prevalent. Examples of such durability include *Sr31* which protected wheat from major losses for over 30 years until the Ug99 outbreak in 1999 (Ayliffe *et al*., 2008; Pretorius *et al*., 2000; Singh *et al*., 2006) and barley *Rpg1*, which has been widely deployed since the 1940s (Brueggeman *et al*., 2002). An alternative strategy is the simultaneous deployment of several *R* genes within a cultivar to prolong *R* gene efficacy in the field. There is no selective advantage for pathogen strains that have mutated to overcome a single *R* gene in such cultivars, thus imposing a barrier to the stepwise evolution of virulence (Dangl *et al*., 2013; Ellis *et al*., 2014; McDonald and Linde, 2002). However, it is difficult to ensure that multiple*R* genes, which may be scattered throughout the genome, remain together in a breeding programme.

None‐the‐less, genetic resistance to cereal rust diseases has been fundamental for crop protection. For more than 100 years, breeders have introgressed resistance into wheat by undertaking wide crosses between wheat and its wild or domesticated relatives. Notable examples include the transfer of the stem rust resistance genes *Sr2* from emmer wheat (*Triticum turgidum* subsp. *dicoccum*; McFadden, 1930), *Sr31*, *Sr50* and *Sr1RS^Amigo^* from rye (Secale cereale; Mago *et al*., 2005b), *Sr24* and *Sr26* from *Thinopyrum ponticum* (Mago *et al*., 2005a), and *Sr36* from *T*.* timopheevi* (McIntosh and Gyarfas, 1971). However, sexual incompatibility and long generation times can impose significant barriers to successful gene introgression (Erickson, 1945). Also, linkage drag of deleterious alleles has hindered the deployment of many *Sr* genes in wheat, *for example*,*Sr22* and *Sr43* due to yellow flour pigmentation and/or reduced yield and delayed heading date (Knott, 1984; Marais, 1992; Niu *et al*., 2014).

In contrast to wheat, where 60 stem rust resistance genes have been described (McIntosh *et al*., 2017; Chen, Guo *et al*., 2018), only ten stem rust resistance genes have been reported in barley; these being *Rpg1* (Brueggeman *et al*., 2002; Powers and Hines, 1933; Steffenson, 1992), *Rpg2* (Case *et al*., 2018; Patterson *et al*., 1957), *Rpg3* (Case *et al*., 2018; Jedel, 1990; Jedel *et al*., 1989), *rpg4* (Jin *et al*., 1994), *Rpg5* (Brueggeman *et al*., 2008; Sun and Steffenson, 2005; Sun *et al*., 1996), *rpg6* (Fetch *et al*., 2009), *Rpg7* (Henningsen and Steffenson, 2018), *rpgBH* (Steffenson *et al*., 1984; Sun and Steffenson, 2005), *RpgU* (Fox and Harder, 1995), and an undesignated one from accession Skinless (Luig, 1957). *Rpg1* is the most widely deployed amongst these genes due to its broad‐spectrum resistance which has remained effective for over 70 years (Brueggeman *et al*., 2002; Steffenson, 1992). However, a recent study showed that this gene is not effective against the Ug99 lineage race TTKSK (Steffenson *et al*., 2017). The *rpg4*‐Mediated Resistance Locus (RMRL) is the most effective gene complex identified in barley against race TTKSK. It consists of two tightly linked loci: RMRL‐1, containing the three genes of *HvRga1*, *Rpg5*, and *HvAdf3*, and RMRL2 (Wang *et al*., 2013). In addition, seedling assays undertaken on two panels of 1,924 and 934 genetically diverse barley cultivars and wild barley accessions (*H. vulgare* subsp. *spontaneum*), showed that more than 95% and 97% of accessions respectively, were susceptible to race TTKSK. Hence, it is important to identify novel sources of resistance to safeguard barley from stem rust (Steffenson *et al*., 2017). Given the limited number of *R* genes available for *Pgt* protection in barley, interspecies *R* gene transfer is a potentially valuable alternative (Wulff and Moscou, 2014).

The majority of *R* genes cloned encode proteins containing nucleotide‐binding and leucine‐rich repeat domains (NLR proteins; Kourelis and van der Hoorn, 2018). Plant genomes typically contain several hundred NLR genes (Baggs *et al*., 2017). NLRs detect the presence of a pathogen by recognising the presence of pathogen effector molecules. This recognition can be direct, although more often it is indirect whereby the NLR (also known as the ‘guard’) recognises the effector‐mediated modification of a host pathogenicity target or a ‘decoy’ of this target, (also known as the ‘guardee’) (van der Hoorn and Kamoun, 2008; Dodds and Rathjen, 2010; Kourelis and van der Hoorn, 2018). In some cases, the decoy pathogenicity target is integrated into the NLR itself—such NLRs (dubbed ‘sensor’ NLRs) often work in concert with a second ‘helper’ NLR which initiates downstream signalling upon activation of the sensor NLR (Cesari *et al*., 2014). NLR proteins that function by either mechanism have been successfully transferred by transgenesis to distantly related, nonsexually compatible species and shown to function in some instances. For example, the *L6* protein of flax (*Linum usitatissimum*, a member of the Linaceae) directly binds a corresponding *AvrL567* effector protein of the flax rust pathogen *Melampsora lini*. When the *L6* gene is co‐expressed with *AvrL567* in *Nicotiana benthamiana* (a member of the Solanaceae) a hypersensitive resistance response is activated (Dodds *et al*., 2004). Similarly, a number of *R* genes that function by guardee recognition have been shown to function upon interspecies transfer, exemplified by the transfer of the *Arabidopsis thaliana* (a Brassicaceae) guard and guardee gene pairs *RPS2* or *RPM1* with *RIN4* (Chung *et al*., 2011; Day *et al*., 2005) and *RPS5* with *PBS1* (Ade *et al*., 2007) into *N*.* benthamiana*. Finally, the Arabidopsis paired *Rrs1/Rps4* sensor/helper NLRs support functional intra‐family transfer within the Brassicaceae as well as inter‐family transfer to the Solanaceae (*N*.* benthamiana* and *Solanum lycopersicum*) and Cucurbitaceae (*Cucumis sativus*) (Narusaka *et al*., 2013).

Transferring *R* genes between species by conventional crossing can be a tedious task due to the extensive backcrossing usually required. However, it is now relatively straightforward to introduce these *R* genes as transgenes by transformation thereby avoiding this breeding requirement. Further advantages of transgenesis include that transfer is not limited to sexually compatible species, there is no linkage drag, and it becomes possible to stack multiple *R* genes at the same locus to ensure co‐inheritance. When transferred between different species and families, these *R* genes can function normally (reviewed in Wulff *et al*., 2011) and agronomically important examples include the *Bs2* gene from pepper (*Capsicum annuum*), which was successfully transferred to tomato (*S. lycopersicum*), another Solanacous species, where it confers resistance to bacterial leaf spot (Tai *et al*., 1999) and *CcRpp1* from pigeonpea (*Cajanus cajan*), which confers resistance to Asian soybean rust when introduced into soybean (*Glycine max*; Kawashima *et al*., 2016).

**Figure 1:**
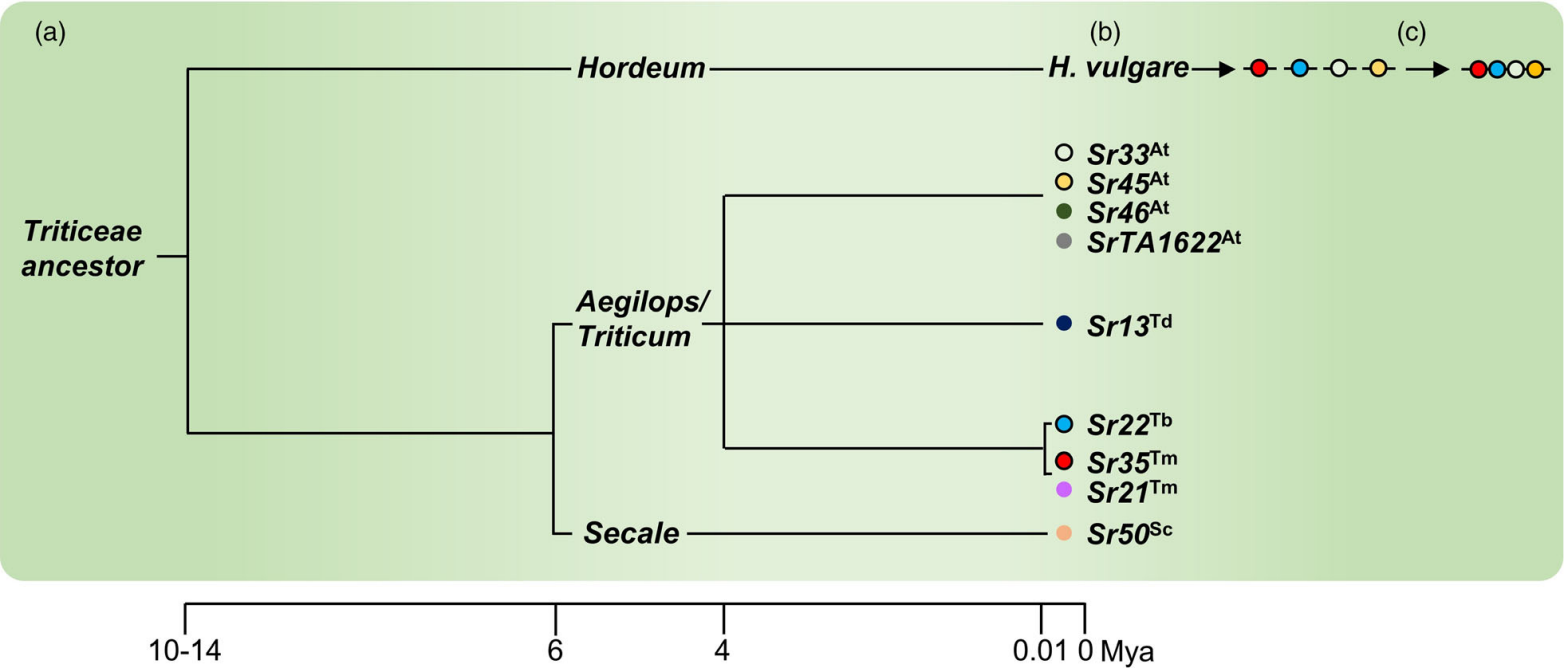
Strategy for improving barley resistance to stem rust with cloned wheat *Sr* genes. (a) Wheat and barley diverged from a common Triticeae ancestor 10‐14 million years ago. (b) Cloned *Sr* genes from wheat, rye and the domesticated and wild relatives of wheat (coloured circles), function when transformed into barley (black outline, this study). (c) The future stacking of multiple cloned *Sr* genes in barley may provide durable resistance to wheat stem rust. At, *Aegilops tauschii*; Td, *Triticum turgidum* ssp. *durum*; Tb, *Triticum boeoticum*; Tm, *Triticum monococcum*; Sc, *Secale cereale*. [Colour figure can be viewed at wileyonlinelibrary.com]

Barley (*H*.*vulgare*) and wheat (*T*.*aestivum*) diverged from a common Triticeae ancestor approximately 10 to 14 million years ago (Schlegel, 2013; Figure 1a). It is therefore likely that wheat NLR genes will function in barley, and that wheat *Sr* genes could be used to improve the resistance of barley to *Pgt*. Nine NLR‐encording *Sr* genes have been cloned so far from wheat or its wild progenitors: *Sr13* from durum wheat (*T*.*turgidum* ssp. *durum*; Zhang *et al*., 2017), *Sr21*, *Sr22* and *Sr35* from *T*.*boeoticum* and *T*.*monococcum* (Chen, Zhang *et al*., 2018; Saintenac *et al*., 2013; Steuernagel *et al*., 2016), *Sr33*, *Sr45*, *Sr46* and *SrTA1662* from *Aegilops tauschii* (Arora *et al*., 2019; Periyannan *et al*., 2013; Steuernagel *et al*., 2016), and *Sr50* from rye (*Secale cereale*; Mago *et al*., 2015; Figure 1b). All these genes confer resistance to the UG99 race group.

In the coming years, it is anticipated that there will be a large increase in the number of cloned *Sr* genes due to the development of rapid *R* gene isolation methods such as TACCA (Thind *et al*., 2017), MutRenSeq (Steuernagel *et al*., 2016), MutChromSeq (Sánchez‐Martín *et al*., 2016) and AgRenSeq (Arora *et al*., 2019). Often the functional testing of *R* gene candidates is delayed by the need to isolate native regulatory sequences and to assemble large binary constructs encoding the *R* gene. This process can be accelerated by substituting regulatory elements from the previously cloned *R* genes, and generating *R* gene constructs using the type IIS restriction endonuclease‐based Golden Gate cloning technique (Engler *et al*., 2008). Further, the incorporation of type IIS restriction sites allows the generation of user‐defined overhangs thereby enabling simultaneous cloning of multiple fragments. This assembly method has dramatically decreased the amount of time required to design and develop gene constructs. However, one major requirement is that the fragments to be assembled must be free from recognition sites of the selected type IIS restriction endonuclease. This requires ‘sequence domestication’ (removal of internal type IIS sites). While the open reading frame can be maintained due to the redundancy in the genetic code, the removal of sites from the regulatory sequences (introns, promoter and terminator) may affect gene expression and function.

In this study, we generated constructs encoding the wheat *Sr22*, *Sr33*, *Sr35* and *Sr45* genes using Golden Gate cloning and transformed these into barley (Figure 1b). The resultant transgenic barley lines showed high levels of resistance to *Pgt*. Future stacking of these *Sr* genes might therefore be used to engineer more durable immunity towards wheat stem rust in barley (Figure 1c).

## Results

### The wheat *Sr22* and *Sr33* genes confer resistance against wheat stem rust in transgenic barley

**Figure 2:**
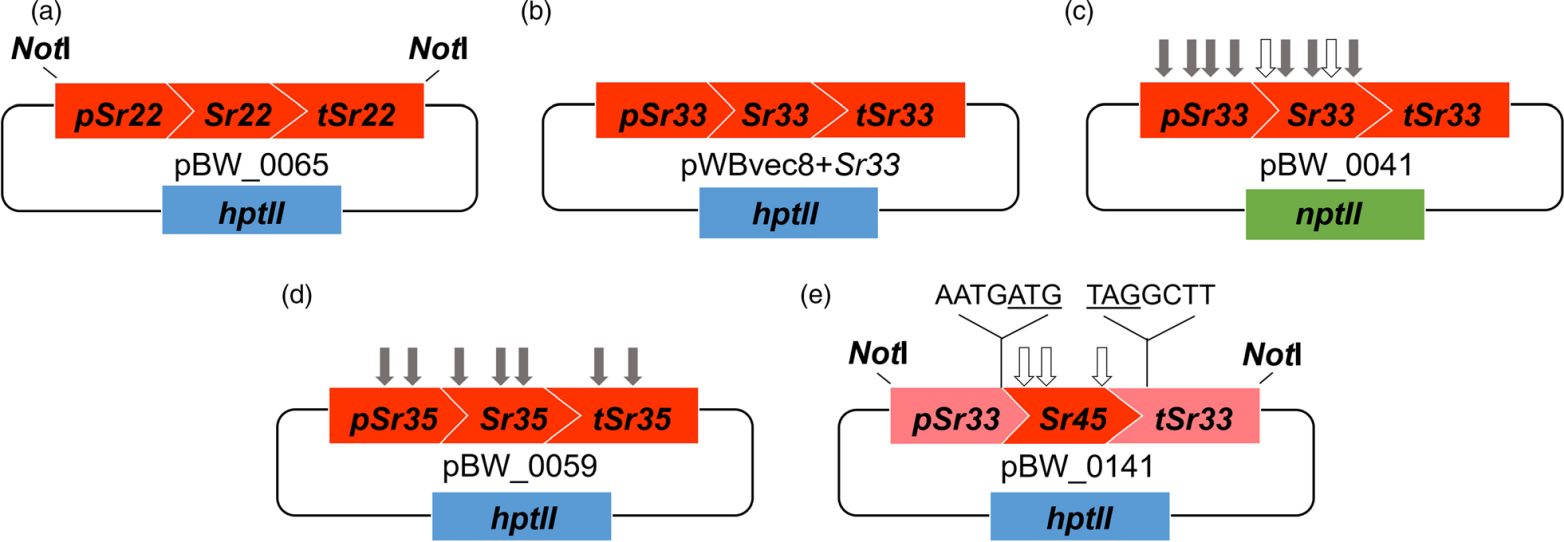
Schematic overview (not shown to scale) of the constructs described in this study. Binary construct containing (a,b) full‐length *Sr22* and *Sr33*, respectively, driven by their 5′ and 3′ native regulatory elements, (c) *Bsa*I and *Bpi*I domesticated full‐length *Sr33^d^* driven by its 5′ and 3′ native regulatory elements, (d) *Bpi*I domesticated full‐length *Sr35^d^* driven by its 5′ and 3′ native regulatory elements and (e) *Bsa*I domesticated *Sr45^d^* driven by 5′ and 3′ *Sr33* regulatory elements. The non‐native four‐nucleotide linker in pBW_0141 was introduced immediately before the start codon (underlined) and immediately after the stop codon (underlined). Grey arrows correspond to the removed *Bpi*I sites and white arrows correspond to the removed *Bsa*I sites. Sites removed in the coding regions were achieved by introducing synonymous mutations. Blue and green rectangles correspond respectively to *hpt*II and *npt*II plant selectable marker genes. [Colour figure can be viewed at wileyonlinelibrary.com]

To determine whether cloned wheat *Sr* genes can function in barley to confer wheat stem rust resistance, barley cultivar (cv.) Golden Promise was transformed with constructs encoding either *Sr22* or *Sr33* via *Agrobacterium*‐mediated transformation. The *Sr22* (9.8 kb) and *Sr33* (7.9 kb) sequences encoded their respective native 5′ and 3′ regulatory sequences and were identical in sequence to the endogenous wheat genes (Figure 2a and b; Table S1). Single copy, hemizygous, primary transgenics were identified amongst T_0_ barley plants by qPCR so that single copy segregating T_1_ or T_2_ families, or homozygous T_2_ lines could be tested in subsequent generations.

**Figure 3:**
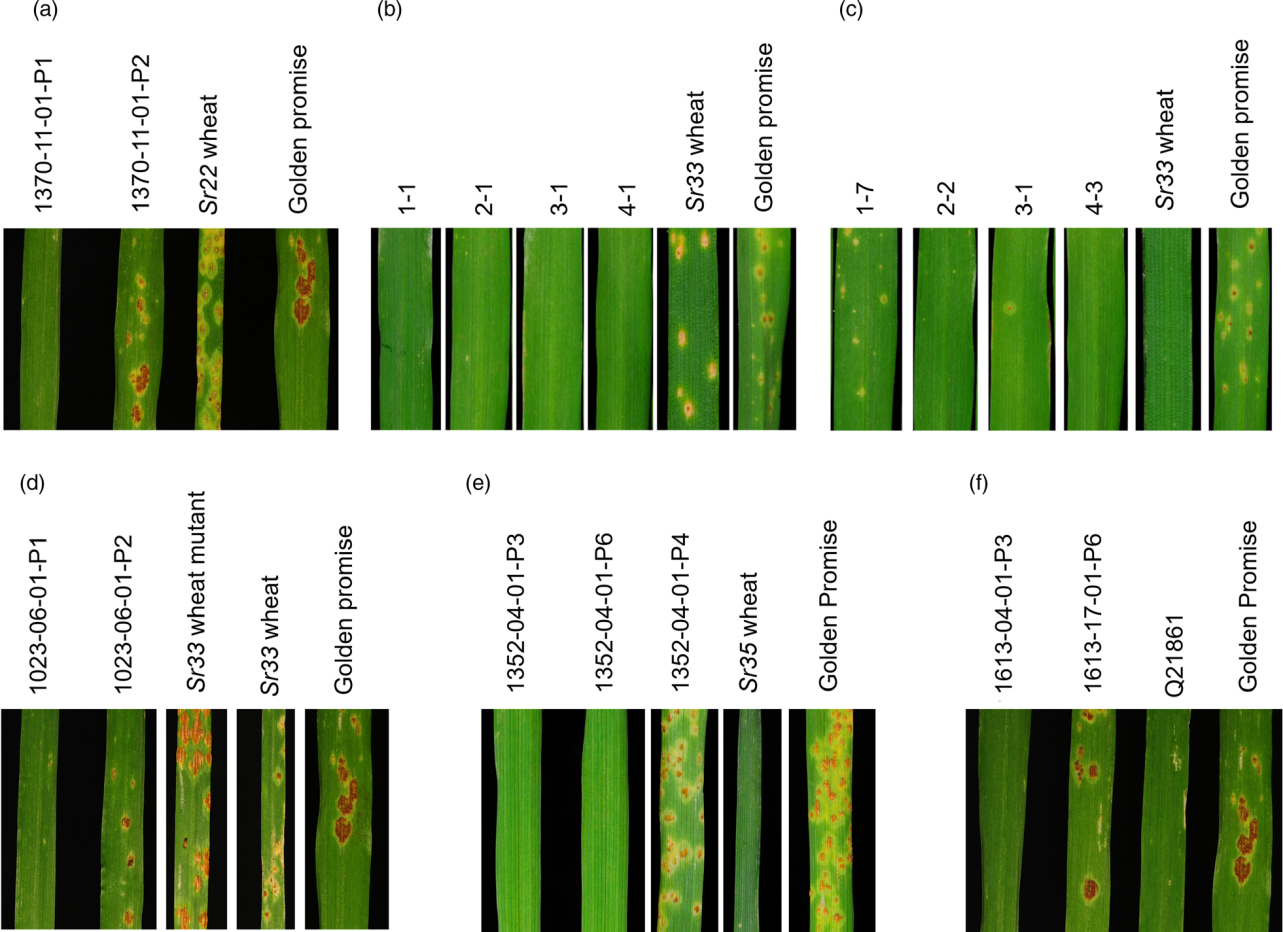
Transgenic *Sr22*,*Sr33*,*Sr33^d^, Sr35^d^* and *Sr45^d^* expression provide resistance against stem rust in barley at the seedling stage. Stem rust infection assays using *Pgt* race (a) MCCFC on *Sr22* T_2_ homozygous transgenics 1370‐11‐01 (P1 and P2) and comparison to resistant *Sr22* wheat and susceptible cv. Golden Promise wild type used in transformation, (b) MCCFC on *Sr33* T_2_ homozygous transgenics 1‐1, 2‐1, 3‐1, 4‐1 and comparison to resistant *Sr33* wheat and susceptible cv. Golden Promise, (c) TKTTF on *Sr33* T_2_ homozygous transgenics 1‐7, 2‐2, 3‐1, 4‐3 and comparison to resistant *Sr33* wheat and susceptible cv. Golden Promise, (d) MCCFC on *Sr33^d^* T_2_ homozygous transgenics 1023‐06‐01 (P1 and P2) and comparison to resistant *Sr33* wheat, susceptible *Sr33* EMS‐induced mutant wheat line and susceptible cv. Golden Promise, (e) TKTTF on *Sr35^d^* T_3_ homozygous transgenics 1352‐04‐01 (P3, P4 and P6), and comparison to resistant *Sr35* wheat and susceptible cv. Golden Promise, and (f) MCCFC on *Sr45^d^* T_2_ homozygous transgenics 1613‐04‐01‐P3 and 1613‐17‐01‐P6 and comparison to resistant check Q21861 and susceptible cv. Golden Promise. The race MCCFC test in panels (a), (d) and (f) were done at the same time with the same Golden Promise susceptible control. [Colour figure can be viewed at wileyonlinelibrary.com]

Four homozygous and three null lines for the *Sr22* transgene were identified from one *Sr22* segregating T_1_ family. The seedlings were inoculated with *Pgt* race MCCFC and all four homozygous lines showed resistance (Figure 3a and Table S2). The homozygous lines showed near immunity to this *Pgt* isolate (Figure 3a and Table S2) whereas null lines and Golden Promise control seedlings all showed extensive *Pgt* growth.

Four T_2_ lines, each derived from an independent transgenic event, were selected that were homozygous for the *Sr33* transgene and T_3_ families (11‐12 seedlings) from each line inoculated with *Pgt* race MCCFC and TKTTF. All seedlings tested showed resistance to both races of *Pgt* (Figure 3b and c; Table S3). In addition to susceptible Golden Promise control seedlings, additional wheat control lines were included. The *Sr33*‐containing cv. Chinese Spring and an EMS‐derived mutant carrying a non‐functional allele of *Sr33* (Periyannan *et al*., 2013) were used as resistant and susceptible controls, respectively, to demonstrate the avirulence of *Pgt* race MCCFC to *Sr33* (Table S3).

### The wheat *Sr33*, *Sr35* and *Sr45* genes can be sequence‐modified but retain gene function

A functional wheat NLR transgene is typically 10 kb in length and consists of 4 kb of 5′ and 3′ regulatory elements, 3 kb of exons and 3 kb of introns. These long, contiguous sequences can be difficult to isolate and verify from a non‐reference hexaploid wheat genome, and their synthesis is expensive. Multi‐segment Golden Gate assembly (Weber *et al*., 2011) was therefore tested as an alternative for rapid and cost‐effective generation of full‐length *Sr* gene constructs using either native or non‐native regulatory sequences. Firstly, the effect of sequence domestication (*i*.*e*. the removal of all Type IIS *Bsa*I and *Bpi*I restriction enzyme sites) was examined on *Sr33* function. Four *Bpi*I sites were removed from the *Sr33* promoter while three *Bpi*I and two *Bsa*I sites were removed from the *Sr33* open reading frame (Figure 2c). Although the *Sr33* open reading frame was faithfully maintained through domestication, there was a risk that the removal of the four *Bpi*I sites in the 5′ regulatory sequence would disrupt gene function. The domesticated full‐length *Sr33* gene (*Sr33^d^*) was transformed into cv. Golden Promise. In total, five homozygous and five null lines were selected from two segregating T_1_ families and infected with *Pgt* race MCCFC. All five homozygous lines conferred resistance while the five null lines showed intermediate to susceptible reactions (Figure 3d and Table S4). These data confirm that *Sr33^d^* encodes functional *Sr33* resistance in spite of the sequence domestication process.

Having demonstrated that the endogenous wheat genes *Sr22* and *Sr33* can provide *Pgt* resistance in barley and that a sequence‐modified version of *Sr33* (*Sr33^d^*) maintains function, two additional domesticated wheat *Sr* genes were developed. A domesticated *Sr35* construct (*Sr35^d^*) was generated by multi‐segment Golden Gate assembly which involved removing seven *Bpi*I sequences from the gene (Figure 2d). In total, eight homozygous and nine null lines were selected from three *Sr35^d^* segregating T_1_ families and tested with *Pgt* race TKTTF. All eight homozygous lines showed a high level of resistance while the nine null lines were all susceptible (Figure 3e and Table S5). A chimeric *Sr45* gene construct (*Sr45^d^*) was also assembled by Golden Gate and consisted of a *Bsa*I domesticated *Sr45* open reading frame flanked by *Sr33* 5′ and 3′ regulatory sequences (Figure 2e). This construct did not require the removal of *Bpi*I sites from *Sr33* regulatory sequences; however, the assembly resulted in the introduction of four additional nucleotides at both the junction between the *Sr33* promoter and start codon of *Sr45* and the termination codon of the *Sr45* ORF and the *Sr33* 3′ regulatory sequence (Figure 2e). In spite of these modifications all seven homozygous lines selected from three *Sr45^d^* segregating T_1_ families showed a very high level of resistance to *Pgt* race MCCFC (Figure 3f and Table S6). These data demonstrate that these wheat NLR genes can be sequence‐modified to facilitate further molecular biological manipulation and that regulatory sequences can be functionally exchanged between NLR genes in some instances.

### Pathogen and race‐specific resistance is maintained by wheat *Sr* genes in barley

To rule out the possibility that these resistant transgenic barley lines are a consequence of an ectopic non‐specific defence reaction, we tested these transgenic plants with the barley leaf rust pathogen *Puccinia hordei*. All *Sr22*, *Sr33*, *Sr33^d^*, *Sr35^d^* and *Sr45^d^* transgenic barley lines, as well as Golden Promise was susceptible or moderately susceptible to *P*.*hordei*. In contrast, a barley control, accession PI584760 carrying *Rph14*, and the wheat *Sr33* line, which is a non‐host of *P*.*hordei*, were both resistant indicating that the barley resistance observed in stem rust infection assays was specific to *Pgt* (Figure S1; Table S7‐S11). Ten *Sr35^d^* transgenic T_1_ families were also tested with *Pgt* race MCCFC (virulent to *Sr35*) including the three T_2_ lines described above that are resistant to *Pgt* race TKTTF. All ten *Sr35^d^* T_1_ families were intermediate to susceptible to *Pgt* race MCCFC (Table S12) indicating that race specificity of this wheat gene is maintained in transgenic barley.

### Expression of wheat *Sr* genes does not affect agronomic traits in barley

**Figure 4:**
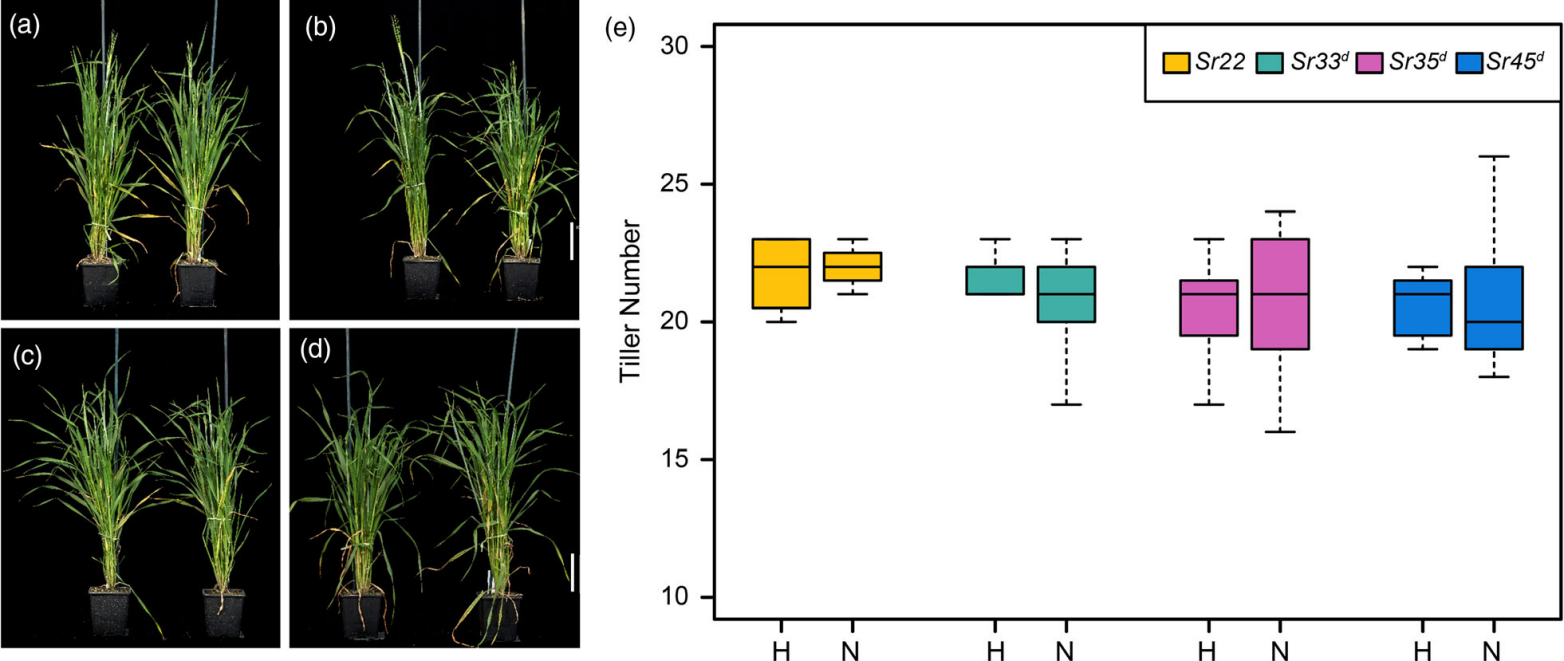
Plant growth and development of *Sr22*, *Sr33^d^*, *Sr35^d^* and *Sr45^d^* representative T_2_ transgenics at 50 d after sowing (DAS). (a) *Sr22* 1370‐11‐01 plant 5, homozygous (right) and plant 15, null (left). (b) *Sr33^d^* 1024‐13‐01 plant 15, homozygous (right) and plant 14, null (left). (c) *Sr35^d^* 1352‐06‐01 plant 4, homozygous (right) and plant 10, null (left). (d) *Sr45^d^* 1613‐04‐01 plant 6, homozygous (right) and plant 2, null (left). Scale bar = 10 cm. (e) Tiller number of *Hordeum vulgare* cv. Golden Promise with and without the presence of transgene. Boxplots are used to indicate variation in tiller number for the biological replicates. Suffixes ‘‐N’ and ‘‐H’ indicate nulls (for absence of the transgene) and homozygous (for presence of the transgene), respectively. [Colour figure can be viewed at wileyonlinelibrary.com]

**Figure 5:**
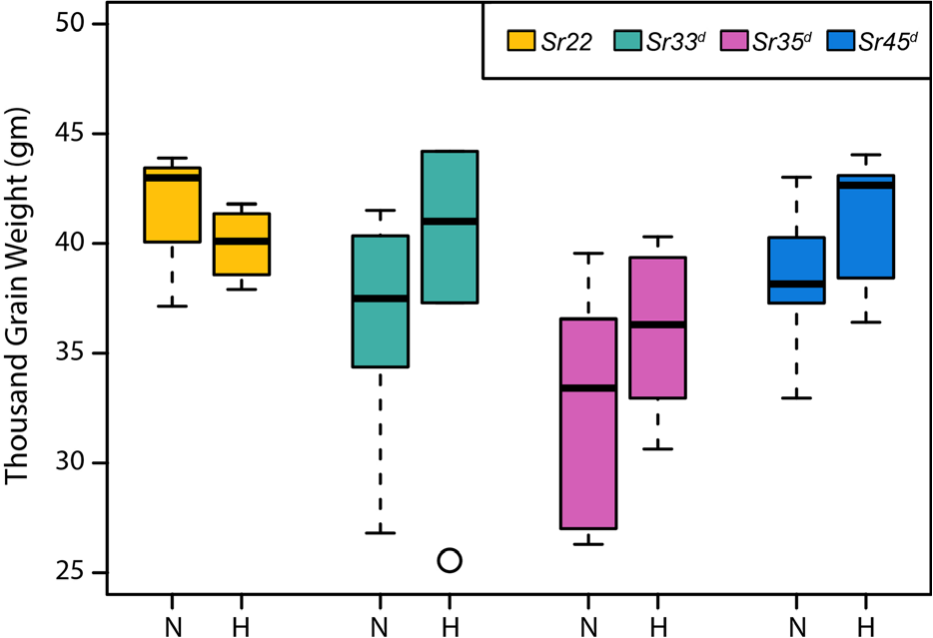
Thousand Grain Weight (TGW) of *Hordeum vulgare* cv. Golden Promise with and without the presence of transgene. Boxplots are used to indicate variation in TGW for the biological replicates. Suffixes ‘‐N’ and ‘‐H’ indicate nulls (for absence of the transgene) and homozygous (for presence of the transgene), respectively. [Colour figure can be viewed at wileyonlinelibrary.com]

To determine whether expression of wheat *Sr* genes in transgenic barley affects agronomic traits, we selected homozygous lines of *Sr22*, *Sr33^d^*, *Sr35^d^* and *Sr45^d^* and compared plant growth and seed characteristics to non‐transgenic Golden Promise in the absence of disease (Figures 4 and 5). Statistical analyses revealed that none of the homozygous *Sr* gene transgenic lines differed significantly from the null lines with regard to the agronomic traits measured including tiller number (Figure 4e and Table S13) thousand grain weight (Figure 5 and Table S14), and growth rate (Figure S2; Table S15‐S18).

## Discussion

Barley is a major food staple in the mountainous areas of Central Asia, Southwest Asia and Northern Africa (Von Bothmer *et al*., 2003). The re‐emergence of wheat stem rust as a major biotic constraint to wheat production also poses a threat to barley production. A recent study revealed very limited resistance to the Ug99 lineage race TTKSK in both cultivated barley and its immediate progenitor *H*.*vulgare* ssp. *spontaneum* (Steffenson *et al*., 2017). This *Pgt* isolate has caused major epidemics in East Africa since 1999. One avenue for improving resistance to stem rust in barley is to utilise diverse genetic resistance from outside the barley gene pool.

*R* genes typically function when transferred from one species to another within the same family (Wulff *et al*., 2011). In this study, the wheat *Sr22*,*Sr33*,*Sr35* and *Sr45* genes were shown to function when transferred to barley and confer race‐specific disease resistance to *Pgt*. Recently, the wheat *Sr35* and *Sr50* have been demonstrated to be functional when transiently expressed in barley (Bolus *et al*., 2020; Saur *et al*., 2019). Other examples of *R* gene transfer in monocots include the introduction of the maize NLR gene *Rxo1* into rice where it confers resistance to bacterial streak disease (Zhao *et al*., 2005) and single‐cell transient expression assays of the barley *Mla6* gene in wheat where it confers *AvrMla6*‐dependent resistance to *Blumeria graminis* f. sp. *hordei* (*Bgh)* (Halterman *et al*., 2001). In concordance, the wheat *Sr22*, *Sr33*,*Sr35* and *Sr45* genes also function in barley, suggesting that the downstream signalling pathway(s) of NLR proteins in wheat and barley has remained conserved since the divergence of these two species 10 to 14 million years ago (Schlegel, 2013).

The functional transfer of these wheat *Sr* genes into barley potentially provides additional sources of stem rust resistance in this recipient species. Interestingly, in most cases, these barley transformants displayed a highly resistant reaction that is stronger than that observed for the endogenous wheat genes. Similarly, when the barley *Rpg1* gene was expressed as a transgene in barley, this also gave rise to a near‐immune reaction (Horvath *et al*., 2003). In contrast to these transgenic barley experiments, near‐immune reactions were extremely rare when large scale screening of wild and cultivated barley lines was undertaken using different *Pgt* races (e.g. Steffenson *et al*., 2017). The increased resistance conferred by these transgenes may be a consequence of elevated expression arising from position effects or alternatively their interaction with a new genetic background in the case of interspecies transfer.

Importantly, we confirmed that race specificity of the *Sr35* gene was maintained in barley. We assume this is also the case for *Sr22*, *Sr33* and *Sr45*, but cannot confirm this due to an absence of *Pgt* races virulent on both Golden Promise and towards these genes. Unlike most barley cultivars, Golden Promise does show resistance to many *Pgt* races which makes *Sr* gene analysis difficult as it is one of the few transformable barley cultivars available. However, for these latter genes, we confirmed that resistance is not a consequence of a non‐specific defence reaction caused by ectopic expression of these genes. All transgenic barley lines tested with *P*.* hordei*, the causal agent of barley leaf rust, were as susceptible as the Golden Promise control lines demonstrating species‐specific resistance conferred by these *Sr* genes. The phylogenetic relatedness of these two fungal pathogen species argues against a generic defence response being activated. Interestingly these data also suggest that there is little conservation of effectors recognised by these wheat *Sr* genes in *P*.* hordei*.

Transgenic plants displaying a resistance phenotype derived from a transgene have been reported to induce ectopic expression of downstream defence genes in the absence of the pathogen (Mindrinos *et al*., 1994; Oldroyd and Staskawicz, 1998), which may affect plant growth and development. Such precocious triggering of defence responses could result from inappropriate interaction between NLR proteins and the pathogenicity targets they guard (Kruger *et al*., 2002). We showed that there was no significant difference between barley transgenic homozygous and null lines of the four *Sr* genes with respect to agronomic traits such as tiller number, growth progression, or thousand grain weight (TGW) (Figures 4 and 5; Figure S2; Table S13‐S18), indicating that ectopic defence activation by the wheat *Sr* genes in barley was absent or minimal. In contrast, expression in barley of the wheat leaf rust resistance gene *Lr34*, which encodes an abscisic acid transporter (Krattinger *et al*., 2019), provided resistance to multiple pathogens (Risk *et al*., 2013), concomitant with severe necrosis and stunted growth (Chauhan *et al*., 2015). In this case, pathogen resistance and necrosis could be uncoupled by co‐expression of a susceptible allele of *Lr34* (Chauhan *et al*., 2015).

In the last few years, many significant improvements have been made in the field of *R* gene cloning. For example, sequence comparison of multiple independently‐derived mutants, facilitated by various genome complexity reduction technologies, for example, NLR exome capture (Steuernagel *et al*., 2016) or chromosome flow sorting (Sánchez‐Martín *et al*., 2016; Thind *et al*., 2017) was used to rapidly clone *Sr22*, *Sr45*,*Pm2* and *Lr22a* from hexaploid wheat. Recently, the requirement of mutagenesis was overcome by combining association genetics with NLR exome capture on a diversity panel of *Ae*.*tauschii* (Arora *et al*., 2019). The resulting application, AgRenSeq, allowed the rapid cloning of *Sr46* and identification of a high confidence candidate for *SrTA1662* (Arora *et al*., 2019). These advances coupled with the recent availability of a wheat reference genome will greatly accelerate *R* gene discovery and cloning (Md. Hatta et al., 2019).

As more wheat *R* genes are cloned, they can be tested in barley using the strategy demonstrated in this paper and, in the case of stem rust potentially provide greater control of the disease in this species. The ability to modify these *R* gene sequences by multi‐segment Golden Gate assembly (Weber *et al*., 2011), even including regulatory elements, and yet maintain gene function will greatly facilitate the manipulation and validation of these genes. Unlike hexaploid wheat, the diploid nature of barley will help contribute to our understanding of the fundamental aspects of wheat stem rust resistance. Its greater amenability to mutagenesis will enable the identification of additional genes required for rust *R* gene function, as well as potential host susceptibility genes. Interestingly a higher proportion of rust *R* genes in barley are recessive (26.3%) compared to wheat (6.7%) (Uauy *et al*., 2017). The cloning of such recessive resistance genes may provide novel fundamental insight into plant pathogen interactions.

NLR genes are not the sole means of generating disease resistant plants. Another approach to improve wheat stem rust resistance in barley is to combine multiple, additive minor effect quantitative trait loci (QTLs). Bi‐parental and genome‐wide association studies (GWAS) have identified QTLs associated with stem rust resistance in barley (Case *et al*., 2018; Mamo, 2013; Sallam *et al*., 2017; Turuspekov *et al*., 2016; Zhou *et al*., 2014). More recently, GWAS on adult plants identified seven novel QTLs conferring adult plant resistance to *Pgt* race QCCJB and a composite of races TTKSK, TTKST, TTKTK and TTKTT, which are all members of the Ug99 lineage (Case *et al*., 2017). The presence of adult plant resistance (APR) genes or minor effect QTLs has been shown to enhance the strength of race‐specific *R* genes (Hiebert *et al*., 2016) and promote their longevity (Brun *et al*., 2010).

Interestingly an APR gene has also been transferred between monocot species by transgenesis and shown to function. The wheat *Lr34* APR gene has been shown to provide resistance against multiple, diverse rust, mildew and blast fungal pathogens in barley, rice, durum wheat, maize and sorghum (Krattinger *et al*., 2016; Rinaldo *et al*., 2017; Risk *et al*., 2013; Schnippenkoetter *et al*., 2017; Sucher *et al*., 2017), although the mechanism of this resistance is as yet unknown. Wide interspecies transfer of functional disease resistance is therefore not limited to NLR genes.

Given that wheat *R* genes function in barley, it is reasonable to expect that barley *R* genes will also function in wheat. Therefore, barley *R* genes conferring resistance to wheat stripe rust (*P*.*striiformis* f. sp. *tritici*) (Dawson *et al*., 2016) might be deployed in wheat to control this disease. However, caution should be taken so that *R* genes transferred from one crop to another are not easily overcome which would potentially facilitate a host jump and create a new disease problem. Ideally, *R* genes with different specificities should be combined as a multi *R* gene stack (Figure 1c), preferably with the inclusion of APR genes. This is likely to confer more durable resistance by delaying the emergence of resistance‐breaking strains of the pathogen (Dangl *et al*., 2013; McDonald and Linde, 2002).

In summary, functional transfer of the *Sr22*, *Sr33*,*Sr35* and *Sr45* genes into barley has created a new source of resistance to stem rust in barley. As more novel rust *R* genes are cloned and shown to function in barley, these could subsequently be deployed in a stack to provide broad‐spectrum resistance and reduce the risk of resistance breakdown. Future field experiments with transgenic barley plants expressing single or multiple *Sr* transgenes will be useful to assess the agronomic value of wheat *Sr* genes in barley cultivation.

## Experimental procedures

### Generation of binary constructs carrying *Sr* genes

To assemble a plant transformation construct containing an *Sr22* expression cassette, a 9855 bp fragment of DNA containing the *Sr22* coding sequence, 2377 bp of 5′ regulatory sequence (i.e. 5′ of the predicted start codon) and 1560 bp of 3′ regulatory sequence (*i*.*e*. 3′ of the STOP codon) was synthesised by a commercial DNA synthesis provider (Life Technologies Ltd) with flanking *Not*I sites. The synthetic DNA was cloned into the *Not*I site of the pVec8 binary vector (Wang *et al*., 1998).

The *Sr33* gene sequence from the binary vector pVecNeo + *Sr33* (Periyannan *et al*., 2013) was introduced into the binary vector pWBvec8 (Steuernagel *et al*., 2016) using *Psp*OMI and *Not*I restriction enzymes. The *Sr33* gene sequence in the later construct pWBvec8 + *Sr33* was proof read using AtM5F1, AtM5R1, AtM5F2, AtM5R2, AtM5F3 and AtM5R3 primers (Periyannan *et al*., 2013).

To generate *Sr33^d^*, a 7854 bp fragment of *Sr33*, including 2381 bp of 5′ and 1405 bp of 3′ regulatory sequence and an 8255 bp fragment of *Sr35^d^*, including 2462 bp of 5′ and 2615 bp of 3′ regulatory sequence was synthesised flanked by a pair of divergent *Bpi*I recognition sites. Prior to synthesis, any recognition sequences for the restriction endonucleases *Bsa*I and *Bpi*I were removed by introducing synonymous mutations in coding sequences and avoiding intron splice junctions. This fragment was simultaneously assembled using Golden Gate cloning (Weber *et al*., 2011) into a level two Golden Gate acceptor plasmid, pAGM4723 (Weber *et al*., 2011), with a hygromycin selectable marker cassette to confer resistance to hygromycin.

A transformation construct for barley containing an *Sr45^d^* expression cassette (with internal *Bsa*I sites removed by introducing synonymous mutations) was constructed as described in Arora *et al*., (2019) and the assembled gene cassette was cloned into the *Not*I site of the pVec8 binary vector. All binary plasmids containing the desired insert were transformed into *Agrobacterium tumefaciens* (strain AGL1) for transformation of barley.

### Barley transformation

*Agrobacterium*‐mediated transformation of *Sr* gene constructs *Sr22*, *Sr33^d^*, *Sr35^d^* and *Sr45^d^* into barley cv. Golden Promise was performed as described in (Hinchliffe and Harwood, 2019). The number of transgene copies of *Sr22*, *Sr33^d^*, *Sr35^d^* and *Sr45^d^* in barley was determined by iDNA Genetics (Norwich, UK) using qPCR as described in Bartlett *et al*., (2008). In brief, a PCR amplicon targeting the neomycin phosphotransferase II (*nptII*) or hygromycin phosphotransferase II (*hptII*) gene (with a 6‐carboxyfluorescein; FAM reporter) and an amplicon targeting a barley internal positive control (IPC, with a 2′‐chloro‐7′phenyl‐1,4‐dichloro‐6‐carboxyfluorescein; VIC reporter) were amplified together in a multiplex reaction (15 min denaturation, 40 cycles of 95 °C for 15 s and 60 °C for 60 s) in an ABI7900 PCR machine. Fluorescence from the FAM and VIC fluorochromes was measured during each 60 °C step, and the cycle threshold (Ct) values obtained. The difference in Ct value between the *nptII* or *hptII* gene and the IPC was used to allocate the assayed samples into groups with the same gene copy number. Plants with a single copy and homozygous for the transgene were selected for phenotyping. The stabilised *Sr22*, *Sr33^d^*, *Sr35^d^* and *Sr45^d^* transgenic barley lines are available from the GRU SeedStor (https://www.seedstor.ac.uk/). Binary vector pWBvec8 + *Sr33* was transformed into cv. Golden Promise using *Agrobacterium*‐mediated transformation as described in Moore *et al*. (2015). Four advanced generation lines, SH1, SH2, SH3 and SH4, were selected as homozygous for the *Sr33* transgene by screening with *Sr33* sequence‐specific primers.

### Functional testing of transgenic barley with *Pgt* races MCCFC and TKTTF

To identify a *Pgt* race which would be virulent on Golden Promise and avirulent on *Sr33* and *Sr45*, we interrogated a panel of 151 *Ae*.* tauschii* accessions which had their NLR repertoires sequenced (Arora *et al*., 2019) by BLAST search with the *Sr33*, *Sr45*, *Sr46* and *SrTA1662* gene sequences (using a ≥99% identity and 100% query coverage cut‐off). We identified one accession that appeared to contain only *Sr33*, and four accessions that appeared to contain only *Sr45*. These accessions were resistant to *Pgt* race MCCFC, a race which was previously shown to be virulent on Golden Promise (Arora *et al*., 2013; Kleinhofs *et al*., 2009), whereas 31 *Ae*.*tauschii* accessions which did not appear to contain any of the aforementioned *Sr* genes were predominantly susceptible or intermediate in their response to MCCFC (Table S19). From this, we concluded that MCCFC is avirulent towards *Sr33* and *Sr45*. We also chose MCCFC for screening the barley *Sr22* transgenics since this gene was previously postulated to be effective against this race with an infection type (IT) of 1; (Rouse and Jin, 2011).

### Wheat rust inoculations and phenotypic evaluations

For the stem rust inoculations, *Sr* barley (*Sr22*,*Sr33^d^*,*Sr35^d^*,*Sr45^d^*) T_1_ or T_2_ plants alongside with the susceptible control cv. Golden Promise was infected with *Pgt* race MCCFC or/and TKTTF 10 days after planting. The inoculated plants were rated for disease response 12–14 days after inoculation as previously described in Yu *et al*. (2017). For *Sr33*, T_2_ plants alongside with the susceptible control cv. Golden Promise was infected with *Pgt* race MCCFC (isolate 59KS19) and TKTTF (isolate 13ETH18‐1). Inoculation, incubation and disease assessment procedures were performed as described previously (Zhang *et al*., 2017). At least 10 plants from each homozygous family were evaluted for *Sr33* and ITs were recorded once, 12 days after inoculation.

For the leaf rust experiment, each cone rack (98 cones × three seeds/cone = 294 plants/cone rack) received 1 mL of inoculum (15 mg spore) of *P*.* hordei* race 4 (Levine and Cherewick, 1952) across the primary leaves of 8–9 day‐old seedlings. *P*.* hordei* isolate 12TX15‐2 was used to inoculate the *Sr33‐*transformed lines. Therefore, each plant received 0.05 mg of urediniospores. To minimise risk of phytotoxicity, the Soltrol 170 oil carrier was evaporated from the leaf surfaces by two hours of gentle fanning under 400‐Watt HPS light bulbs. Inoculated seedlings were incubated at 22 °C inside mist chambers with 100% relative air humidity provided by a household ultrasonic humidifier for 18 h. Post inoculation, plants were moved to a greenhouse running a 16‐hour day length with a night temperature of 15 °C and a day temperature of 20 °C. The resistant control used in the barley leaf rust infection assays was PI584760, which carries the gene *Rph14* (Martin *et al*., 2020).

Disease phenotypes (*i*.*e*. infection types (ITs) were scored twice for each experiment: first at 10 and then at 12 days post inoculation (dpi) as previously described in Park et al. (2015).

### Agronomic trait measurement

Segregating T_1_ progeny derived from single copy (hemizygous) plants were sown in a glasshouse fitted with Heliospectra LED lights positioned to give a light intensity of 303 ± 68 μmol/m^2^/s at bench height and 467 ± 133 μmol/m^2^/s at canopy height (Table S20) and set to a 22 h light, 2 h dark growth ‘speed breeding’ regime as previously described (Ghosh et al., 2018). The leaves of three‐week old plants were sampled for DNA extraction and qPCR (Bartlett *et al*. (2008) to distinguish between homozygous transgenic, hemizygous and nulls. The homozygous and null plants were potted up for further analysis. Tiller number (Figure 4e and Table S13), thousand grain weight (Figure 5; Table S14) and timing of progression to various growth stages including 3‐leaf stage, node development, awn‐peep, spike emergence, anthesis or grain dough stage (Figure S2; Tables S15‐S18) was measured. A Welch’s *t*‐test was conducted to determine if there were any significant differences between transgenics and nulls.

## Conflicts of interest statement

The authors declare no conflict of interest.

## Author contributions

MAMdH, MS, RM, GY, NP and MA designed and generated *Sr22*, *Sr33^d^*, *Sr35^d^* and *Sr45^d^* constructs. MAMdH, MA and WH performed *Sr22*, *Sr33^d^*, *Sr35^d^* and *Sr45^d^* transformation. OM, MR and BJS phenotyped *Sr22*, *Sr33^d^*, *Sr35^d^* and *Sr45^d^* transgenics. SC, DB, XX, TR, RM and MA generated the *Sr33* construct, performed transformation and selected homozygous lines while MNR phenotyped the transgenics. SA and BS performed *Ae*.*tauschii* sequence analysis. SG and SA selected homozygous lines and performed experiments to capture growth and agronomic traits. BBHW, SKP, BJS, EL, NP and WH conceived and designed study. MAMdH drafted manuscript with input from BBHW, BJS, MA, SP and NP. All authors read and approved the final manuscript.

## Supporting information


**Figure S1** Leaf rust infection assays with *P*.*hordei* race 4 on *Sr22*, *Sr33*, *Sr33^d^*, *Sr35^d^* and *Sr45^d^* representative T_1_ and T_2_ transgenics at the seedling stage.
**Figure S2** Timeline of growth stages (expressed as Days After Sowing) for *Hordeum vulgare* cv. Golden Promise with and without the presence of (a) *Sr22* (b) *Sr33^d^* (c) *Sr35^d^* (d) *Sr45^d^*. Boxplots indicate variation in timelines for the biological replicates. Suffixes ‘‐N’ and ‘‐H’ indicate nulls (for absence of the transgene) and homozygous (for presence of the transgene), respectively. Growth stages measured for the first tiller according to the Zadoks’ Scale (Zadoks *et al*., 1974).
**Table S1** List of binary constructs carrying *Sr* gene.
**Table S2** Stem rust infection assays with *Pgt* race MCCFC on *Sr22* T_2_ homozygous lines.
**Table S3** Stem rust infection assays with *Pgt* races MCCFC and TKTTF on *Sr33* T_2_ homozygous lines.
**Table S4** Stem rust infection assays with *Pgt* race MCCFC on *Sr33^d^* T_2_ homozygous lines.
**Table S5** Stem rust infection assays with *Pgt* race TKTTF on *Sr35^d^* T_3_ homozygous lines.
**Table S6** Stem rust infection assays with *Pgt* race MCCFC on *Sr45^d^* T_2_ homozygous lines.
**Table S7** *Puccinia hordei* race 4 infection assays on *Sr22* T_2_ families.
**Table S8** *Puccinia hordei* race 4 infection assays on *Sr33* T_2_ homozygous lines.
**Table S9** *Puccinia hordei* race 4 infection assays with on *Sr33^d^* T_2_ families.
**Table S10** *Puccinia hordei* race 4 infection assays on *Sr35^d^* T_2_ families.
**Table S11** *Puccinia hordei* race 4 infection assays on *Sr45^d^* T_2_ families.
**Table S12** Stem rust infection assays with *Pgt* race MCCFC on *Sr35* T_1_ families.
**Table S13** Tiller number of *Hordeum vulgare* cv. Golden Promise with and without the presence of transgene.
**Table S14** Thousand Grain Weight (TGW) of *Hordeum vulgare* cv. Golden Promise with and without the presence of transgene.
**Table S15** Development stages of *Sr22* transgenics and nulls. Values indicated are expressed as days after sowing (DAS).
**Table S16** Development stages of *Sr33^d^* transgenics and nulls. Values indicated are expressed as days after sowing (DAS).
**Table S17** Development stages of *Sr35^d^* transgenics and nulls. Values indicated are expressed as days after sowing (DAS).
**Table S18** Development stages of *Sr45^d^* transgenics and nulls. Values indicated are expressed as days after sowing (DAS).
**Table S19** Functional testing of *Sr33* and *Sr45* with *Pgt* race MCCFC.
**Table S20** Photosynthetic photon flux density (PPFD) measurements for the LED‐supplemented glasshouse. PPFD was measured in μmol/m^2^/s at a central location using an UPRTek MK350S spectrophotometer and associated uSpectrum software (UPRTek, Taiwan). Values are the mean of eighteen measurements ± the standard deviation taken in a metre square area under a light fixture. Plants were rotated around on a weekly basis.
