# Supplementary material for: The wheat Sr22, Sr33, Sr35 and Sr45 genes confer resistance against stem rust in barley
Source: Plant Biotechnol J. 2020 Sep 6;19(2):273–84. doi: 10.1111/pbi.13460 (PMC7868974; doi:10.1111/pbi.13460)
Supplement: Supplementary file 1 — Figure S1 Leaf rust infection assays with P.hordei race 4 on Sr22, Sr33, Sr33d , Sr35d and Sr45d representative T1 and T2 transgenics at the seedling stage. Figure S2 Timeline of growth stages (expressed as Days After Sowing) for Hordeum vulgare cv. Golden Promise with and without the presence of (a) Sr22 (b) Sr33d (c) Sr35d (d) Sr45d . Boxplots indicate variation in timelines for the biological replicates. Suffixes ‘‐N’ and ‘‐H’ indicate nulls (for absence of the transgene) and homozygous (for presence of the transgene), respectively. Growth stages measured for the first tiller according to the Zadoks’ Scale (Zadoks et al., 1974). Table S1 List of binary constructs carrying Sr gene. Table S2 Stem rust infection assays with Pgt race MCCFC on Sr22 T2 homozygous lines. Table S3 Stem rust infection assays with Pgt races MCCFC and TKTTF on Sr33 T2 homozygous lines. Table S4 Stem rust infection assays with Pgt race MCCFC on Sr33d T2 homozygous lines. Table S5 Stem rust infection assays with Pgt race TKTTF on Sr35d T3 homozygous lines. Table S6 Stem rust infection assays with Pgt race MCCFC on Sr45d T2 homozygous lines. Table S7 Puccinia hordei race 4 infection assays on Sr22 T2 families. Table S8 Puccinia hordei race 4 infection assays on Sr33 T2 homozygous lines. Table S9 Puccinia hordei race 4 infection assays with on Sr33d T2 families. Table S10 Puccinia hordei race 4 infection assays on Sr35d T2 families. Table S11 Puccinia hordei race 4 infection assays on Sr45d T2 families. Table S12 Stem rust infection assays with Pgt race MCCFC on Sr35 T1 families. Table S13 Tiller number of Hordeum vulgare cv. Golden Promise with and without the presence of transgene. Table S14 Thousand Grain Weight (TGW) of Hordeum vulgare cv. Golden Promise with and without the presence of transgene. Table S15 Development stages of Sr22 transgenics and nulls. Values indicated are expressed as days after sowing (DAS). Table S16 Development stages of Sr33d transgenics and nulls. Values indicated [file PBI-19-273-s001.docx]

# Supporting information

##


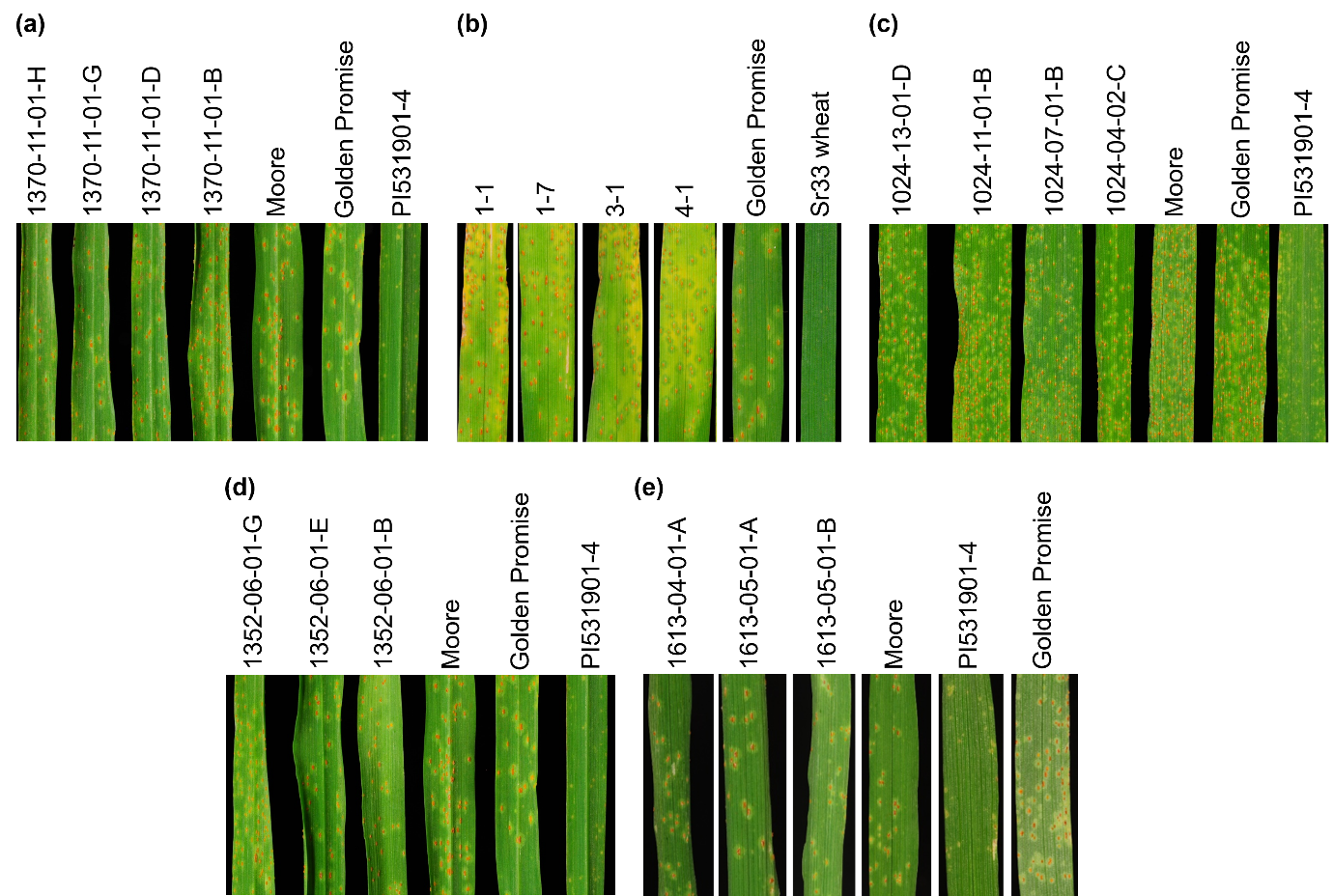


**Figure S1** Leaf rust infection assays with *P. hordei* race 4 on *Sr22*, *Sr33*, *Sr33^d^*, *Sr35^d^*, and *Sr45^d^* representative T_1_ and T_2_ transgenics at the seedling stage. (a) *Sr22* transgenic 1370-11-01-H, 1370-11-01-G, 1370-11-01-D, 1370-11-01-B and comparison to the susceptible control cv Moore and Golden Promise, and the resistant control PI584760. (b) *Sr33* transgenic 1-1, 1-7, 3-1, 4-1 and comparison to the susceptible cv Golden Promise, and the resistant control wheat *Sr33*. (c) *Sr33^d^* transgenic 1024-13-01-D, 1024-11-01-B, 1024-07-01-B, 1024-04-02-C and comparison to the susceptible control cvs Moore and Golden Promise, and the resistant control PI584760. (d) *Sr35^d^* transgenic 1352-06-01-G, 1352-06-01-E, 1352-06-01-B and comparison to the susceptible control cultivars Moore and Golden Promise, and the resistant control PI584760. (e) *Sr45^d^* transgenic 1613-04-01-A, 1613-05-01-A, 1613-05-01-B and comparison to the susceptible control cultivars Moore and Golden Promise, and the resistant control PI584760.


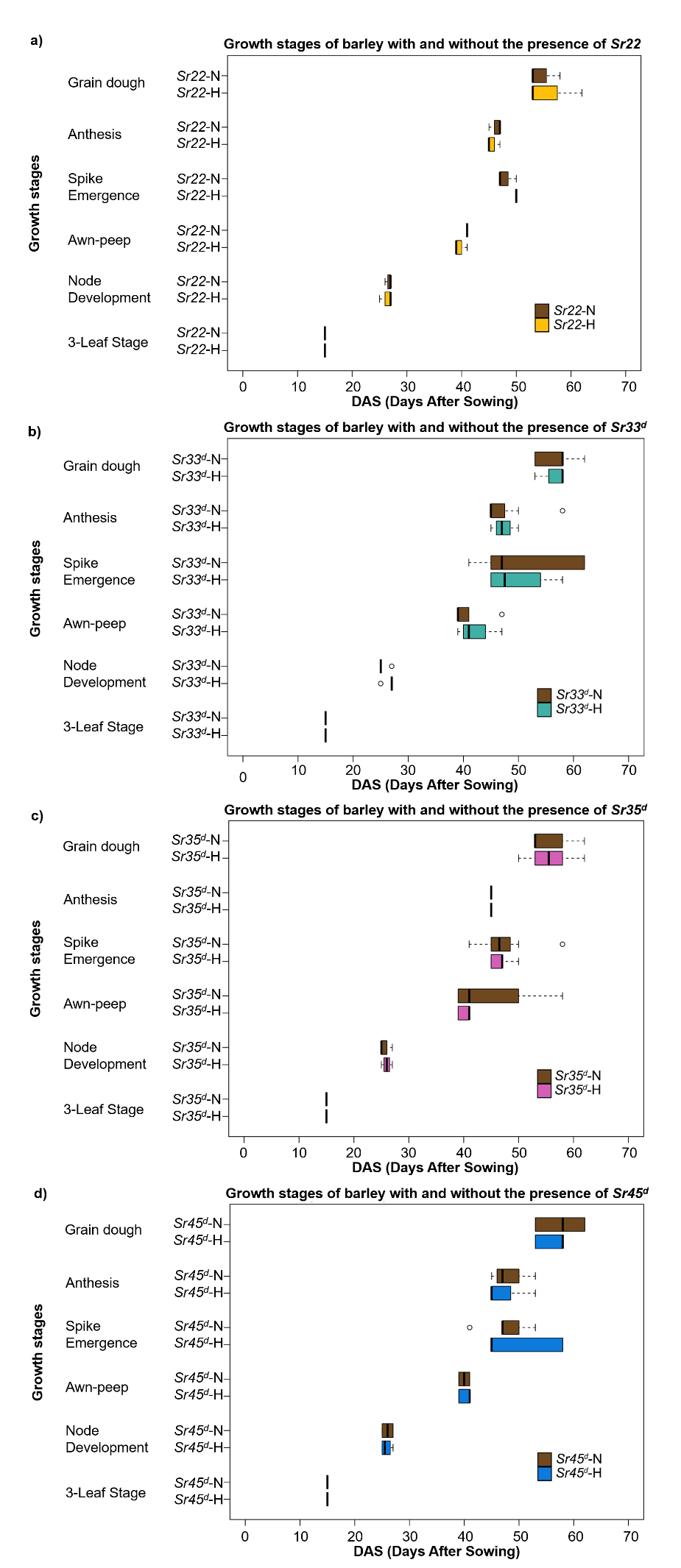


**Figure S2** Timeline of growth stages (expressed as Days After Sowing) for *Hordeum vulgare* cv. Golden Promise with and without the presence of (a) *Sr22* (b) *Sr33^d^* (c) *Sr35^d^* (d) *Sr45^d^*. Boxplots are used to indicate variation in timelines for the biological replicates. Suffixes “-N” and “-H” indicate nulls (for absence of the transgene) and homozygous (for presence of the transgene), respectively. Growth stages measured for the first tiller according to the Zadoks’ Scale (Zadoks et al., 1974).

**Table S1** List of binary constructs carrying Sr gene.

| **Name** | **Gene** | **Binary vector** | **Bacterial Resistance** | **Plant Resistance** | **Regulatory elements** | **Addgene #** |
| --- | --- | --- | --- | --- | --- | --- |
| pBW_0065 | *Sr22* | pVec8 | Spectinomycin | *hptII* | Native | 102817 |
| pWBvec8+*Sr33* | *Sr33* | pVec8 | Spectinomycin | *hptII* | Native | SP^†^ |
| pBW_0041 | *Sr33^d^* | pAGM4723 | Kanamycin | *nptII* | Native | NP^‡^ |
| pBW_0059^§^ | *Sr35^d^* | pAGM4723 | Kanamycin | *hptII* | Native | 102819 |
| pBW_0141^¶^ | *Sr45^d^* | pVec8 | Spectinomycin | *hptII* | *Sr33* promoter and terminator | 102820 |

^†^Available from Sambasivam Periyannan upon request.

^‡^Available from Nicola Patron upon request.

^§^RepA protein has a P65S mutation and a deletion of 145 bp in the backbone.

^¶^T-DNA inserted in the opposite orientation.

**Table S2** Stem rust infection assays with Pgt race MCCFC on Sr22 T_2_ homozygous lines.

| Plant ID | Line | Gene copy | IT1 | IT2 | IT3 | IT4 | IT5 | IT6 |
| --- | --- | --- | --- | --- | --- | --- | --- | --- |
| 1370-11-01-P1 | *Sr22*_T_2_ | 2 | 0 | 0 | 0 | 0 | 0 | 0 |
| 1370-11-01-P3 | *Sr22*_T_2_ | 2 | 0 | 0 | 0 | 0 | 0 | 0 |
| 1370-11-01-P5 | *Sr22*_T_2_ | 2 | 0 | 0 | 0 | 0 | 0 | 0 |
| 1370-11-01-P8 | *Sr22*_T_2_ | 2 | 0 | 0 | 0 | 0 | 0 | 0 |
| 1370-11-01-P2 | *Sr22*_T_2_ | 0 | NA^†^ | 3^-^,2 | 3^-^,2 | 3^-^,2 | 3^-^,2 | 3^-^,2 |
| 1370-11-01-P15 | *Sr22*_T_2_ | 0 | 3^-^,2 | 3^-^,2 | 3^-^,2 | 3^-^,2 | 3^-^,2 | 3^-^,2 |
| 1370-11-01-P16 | *Sr22*_T_2_ | 0 | 3^-^,2 | 3^-^,2 | 3^-^,2 | 3^-^,2 | 3^-^,2 | 3^-^,2 |
| SwSr22 T.B/Wheat Sr22 | *Sr22* wheat |  | 2 | 22^-^, | 2 | 22^-^, | 22^-^, | 2 |
| GP-2015 | Golden Promise |  | 3^-^,2 | 3^-^,2 | 3^-^,2 | 3^-^,2 | 3^-^,2 | 3^-^,2 |
| GP-Saint Paul 2012 | Golden Promise |  | 3^-^,2 | 3^-^,2 | 3^-^,2 | 3^-^,2 | 3^-^,2 | 3^-^,2 |
| McNair | Susceptible control |  | 4 | 4 | 4 | 4 | 4 | 4 |

^†^Not assayed.

**Table S3** Stem rust infection assays with Pgt races MCCFC and TKTTF on Sr33 T_2_ homozygous lines.

| ID | MCCFC | TKTTF |
| --- | --- | --- |
| 1‒1 | 0; | ;12- |
| 1‒2 | 0; | 0;1 |
| 1‒3 | 0; | 0;1 |
| 1‒4 | 0; | 0; |
| 1‒5 | 0; | 0;1- |
| 1‒6 | 0; | 0; |
| 1‒7 | 0; | 0;1- |
| 1‒8 | 0;1 | 0; |
| 1‒9 | 0; | 0;1 |
| 1‒10 | 0;1- | 0; |
| 1‒11 | 0; | 01- |
| 1‒12 | 0; | NA^†^ |
| 2‒1 | 0; | 0; |
| 2‒2 | 0; | 0; |
| 2‒3 | 0; | 0; |
| 2‒4 | 0; | 0; |
| 2‒5 | 0; | 0; |
| 2‒6 | 0; | 0; |
| 2‒7 | 0; | 0; |
| 2‒8 | 0; | 0; |
| 2‒9 | 0; | 0; |
| 2‒10 | 0; | 0; |
| 2‒11 | 0; | 0; |
| 2‒12 | 0; | NA |
| 3‒1 | 0; | 0;1 |
| 3‒2 | 0; | 0; |
| 3‒3 | 0; | 0; |
| 3‒4 | 0; | 0; |
| 3‒5 | 0; | 0; |
| 3‒6 | 0; | 0; |
| 3‒7 | 0;1- | 0;1 |
| 3‒8 | 0;1 | 0;1- |
| 3‒9 | 0; | 0; |
| 3‒10 | 0;1 | 0; |
| 3‒11 | 0; | 0; |
| 3‒12 | 0; | 0; |
| 4‒1 | 0;1- | 0; |
| 4‒2 | 0; | 0; |
| 4‒3 | 0; | 0; |
| 4‒4 | 0; | 0;1 |
| 4‒5 | 0; | 0; |
| 4‒6 | 0; | 0; |
| 4‒7 | 0; | 0; |
| 4‒8 | 0; | 0; |
| 4‒9 | 0; | 0; |
| 4‒10 | 0; | 0; |
| 4‒11 | 0; | 0; |
| 4‒12 | 0; | 0; |
| Golden Promise | 23- | 23-; |
| Wheat *Sr33* | ;1 | ;1 |

^†^Not assayed.

**Table S4** Stem rust infection assays with Pgt race MCCFC on Sr33^d^ T_2_ homozygous lines.

| Plant ID | Line | Gene copy | IT1 | IT2 | IT3 | IT4 | IT5 | IT6 |
| --- | --- | --- | --- | --- | --- | --- | --- | --- |
| 1023-06-01-P1 | *Sr33^d^*_T_2_ | 2 | 0 | 0 | 0 | 0 | 0 | 0 |
| 1023-06-01-P5 | *Sr33^d^*_T_2_ | 2 | 0 | 0 | 0 | 0 | 0 | 0 |
| 1024-13-01-P2 | *Sr33^d^*_T_2_ | 2 | 0 | 0 | 0 | 0 | 0 | 0 |
| 1024-13-01-P11 | *Sr33^d^*_T_2_ | 2 | 0 | 0 | 0 | 0 | 0 | 0 |
| 1024-13-01-P15 | *Sr33^d^*_T_2_ | 2 | 0 | 0 | 0 | 0 | 0 | 1 |
| 1023-06-01-P2 | *Sr33^d^*_T_2_ | 0 | 3^-^,2 | 3^-^,2 | 3^-^,2 | 3^-^,2 | 3^-^,2 | 3^-^,2 |
| 1023-06-01-P10 | *Sr33^d^*_T_2_ | 0 | 3^-^,2 | 3^-^,2 | 3^-^,2 | 3^-^,2 | 3^-^,2 | 3^-^,2 |
| 1024-13-01-P4 | *Sr33^d^*_T_2_ | 0 | 3^-^,2 | 3^-^,2 | 3^-^,2 | 3^-^,2 | 3^-^,2 | 3^-^,2 |
| 1024-13-01-P8 | *Sr33^d^*_T_2_ | 0 | 11^+^, | 11^+^, | 3^-^,2 | 3^-^,2 | 11^+^, | 11^+^, |
| 1024-13-01-P14 | *Sr33^d^*_T_2_ | 0 | 3^-^,2 | 3^-^,2 | 3^-^,2 | 3^-^,2 | 3^-^,2 | 3^-^,2 |
| TA_01046 | *Sr33* wheat |  | 11^+^ | NA^†^ | NA | NA | NA | NA |
| TA_01048 | *Sr33* wheat mutant |  | 33^+^ | NA | NA | NA | NA | NA |
| GP-2015 | Golden Promise |  | 3^-^,2 | 3^-^,2 | 3^-^,2 | 3^-^,2 | 3^-^,2 | 3^-^,2 |
| GP-Saint Paul 2012 | Golden Promise |  | 3^-^,2 | 3^-^,2 | 3^-^,2 | 3^-^,2 | 3^-^,2 | 3^-^,2 |
| McNair | Susceptible control |  | 4 | 4 | 4 | 4 | 4 | 4 |

^†^Not assayed.

**Table S5** Stem rust infection assays with Pgt race TKTTF on Sr35^d^ T_3_ homozygous lines.

| Plant ID | Line | Gene copy | IT |
| --- | --- | --- | --- |
| 1352-04-01-P3 | *Sr35^d^*_T_3_ | 2 | 0 |
| 1352-04-01-P6 | *Sr35^d^*_T_3_ | 2 | 0 |
| 1352-04-01-P7 | *Sr35^d^*_T_3_ | 2 | 0 |
| 1352-04-01-P10 | *Sr35^d^*_T_3_ | 2 | 0 |
| 1352-04-02-P2 | *Sr35^d^*_T_3_ | 2 | 0 |
| 1352-04-02-P4 | *Sr35^d^*_T_3_ | 2 | 0 |
| 1352-04-02-P15 | *Sr35^d^*_T_3_ | 2 | 0 |
| 1352-06-01-P4 | *Sr35^d^*_T_3_ | 2 | 0 |
| 1352-04-01-P4 | *Sr35^d^*_T_3_ | 0 | 3 |
| 1352-04-01-P9 | *Sr35^d^*_T_3_ | 0 | 3 |
| 1352-04-01-P16 | *Sr35^d^*_T_3_ | 0 | 3 |
| 1352-04-02-P1 | *Sr35^d^*_T_3_ | 0 | 3 |
| 1352-04-02-P3 | *Sr35^d^*_T_3_ | 0 | 3 |
| 1352-04-02-P8 | *Sr35^d^*_T_3_ | 0 | 3 |
| 1352-06-01-P5 | *Sr35^d^*_T_3_ | 0 | 3 |
| 1352-06-01-P10 | *Sr35^d^*_T_3_ | 0 | 3 |
| 1352-06-01-P11 | *Sr35^d^*_T_3_ | 0 | 3 |
| G2919 | *Sr35* wheat |  | 0;1^-^ |
| GP-2015 | Golden Promise |  | 3 |
| GP-Saint Paul 2012 | Golden Promise |  | 3 |
| McNair | Susceptible control |  | 4 |

**Table S6** Stem rust infection assays with Pgt race MCCFC on Sr45^d^ T_2_ homozygous lines.

| Plant ID | Line | Gene copy | IT1 | IT2 | IT3 | IT4 | IT5 | IT6 |
| --- | --- | --- | --- | --- | --- | --- | --- | --- |
| 1613-04-01-P3 | *Sr45^d^*_T_2_ | 2 | 0 | 0 | 0 | 0 | 0 | 0 |
| 1613-04-01-P6 | *Sr45^d^*_T_2_ | 2 | 0 | 0 | 0 | 0 | 0 | 0 |
| 1613-05-01-P4 | *Sr45^d^*_T_2_ | 2 | 0 | 0 | 0 | 0 | 0 | 0 |
| 1613-05-01-P10 | *Sr45^d^*_T_2_ | 2 | 0 | 0 | 0 | 0 | 0 | 0 |
| 1613-17-01-P9 | *Sr45^d^*_T_2_ | 2 | 0 | 0 | 0 | 0 | 0 | 0 |
| 1613-17-01-P12 | *Sr45^d^*_T_2_ | 2 | 0 | 0 | 0 | 0 | 0 | 0 |
| 1613-17-01-P3 | *Sr45^d^*_T_2_ | 2 | 0 | 0 | 0 | 0 | 0 | 0 |
| 1613-04-01-P2 | *Sr45^d^*_T_2_ | 0 | 3^-^,2 | 3^-^,2 | 3^-^,2 | 3^-^,2 | 3^-^,2 | 3^-^,2 |
| 1613-04-01-P7 | *Sr45^d^*_T_2_ | 0 | 3^-^,2 | 3^-^,2 | 3^-^,2 | 3^-^,2 | 3^-^,2 | 3^-^,2 |
| 1613-05-01-P2 | *Sr45^d^*_T_2_ | 0 | 3^-^,2 | 3^-^,2 | 3^-^,2 | 3^-^,2 | 3^-^,2 | 3^-^,2 |
| 1613-05-01-P3 | *Sr45^d^*_T_2_ | 0 | 3^-^,2 | 3^-^,2 | 3^-^,2 | 3^-^,2 | 3^-^,2 | 3^-^,2 |
| 1613-05-01-P16 | *Sr45^d^*_T_2_ | 0 | 3^-^,2 | 3^-^,2 | 3^-^,2 | 3^-^,2 | 3^-^,2 | 3^-^,2 |
| 1613-17-01-P4 | *Sr45^d^*_T_2_ | 0 | 3^-^,2 | 3^-^,2 | 3^-^,2 | 3^-^,2 | 3^-^,2 | 3^-^,2 |
| 1613-17-01-P6 | *Sr45^d^*_T_2_ | 0 | 3^-^,2 | 3^-^,2 | 3^-^,2 | 3^-^,2 | 3^-^,2 | 3^-^,2 |
| 1613-17-01-P8 | *Sr45^d^*_T_2_ | 0 | 3^-^,2 | 3^-^,2 | 3^-^,2 | 3^-^,2 | 3^-^,2 | 3^-^,2 |
| 1613-17-01-P10 | *Sr45^d^*_T_2_ | 0 | 3^-^,2 | 3^-^,2 | 3^-^,2 | 3^-^,2 | 3^-^,2 | 3^-^,2 |
| Q21861 | Resistant control |  | 00; | 00; | 00; | 00; | 00; | 00; |
| GP-2015 | Golden Promise |  | 3^-^,2 | 3^-^,2 | 3^-^,2 | 3^-^,2 | 3^-^,2 | 3^-^,2 |
| GP-Saint Paul 2012 | Golden Promise |  | 3^-^,2 | 3^-^,2 | 3^-^,2 | 3^-^,2 | 3^-^,2 | 3^-^,2 |
| McNair | Susceptible control |  | 3 | 3 | 3 | 3 | 3 | 3 |

**Table S7** Puccinia hordei race 4 infection assays on Sr22 T_2_ families.

| Line ID | Plant ID | Line | Infection type |
| --- | --- | --- | --- |
| 1370-01-01 | C | *Sr22*_T_2__leafrust | 3^+^ |
| 1370-01-01 | D | *Sr22*_T_2__leafrust | 3^+^ |
| 1370-01-01 | E | *Sr22*_T_2__leafrust | 3^+^ |
| 1370-01-01 | F | *Sr22*_T_2__leafrust | 3 |
| 1370-01-01 | G | *Sr22*_T_2__leafrust | 3 |
| 1370-01-01 | H | *Sr22*_T_2__leafrust | 3 |
| 1370-11-01 | A | *Sr22*_T_2__leafrust | 3 |
| 1370-11-01 | B | *Sr22*_T_2__leafrust | 33^+^ |
| 1370-11-01 | C | *Sr22*_T_2__leafrust | 3^+^ |
| 1370-11-01 | D | *Sr22*_T_2__leafrust | 3^+^ |
| 1370-11-01 | E | *Sr22*_T_2__leafrust | 3^+^ |
| 1370-11-01 | F | *Sr22*_T_2__leafrust | 3^+^ |
| 1370-11-01 | G | *Sr22*_T_2__leafrust | 3^+^ |
| 1370-11-01 | H | *Sr22*_T_2__leafrust | 3^-^,2 |
| 1372-08-01 | A | *Sr22*_T_2__leafrust | 3 |
| 1372-08-01 | B | *Sr22*_T_2__leafrust | 3^+^ |
| 1372-08-01 | C | *Sr22*_T_2__leafrust | 3^+^ |
| 1372-08-01 | D | *Sr22*_T_2__leafrust | NA^†^ |
| 1372-08-01 | E | *Sr22*_T_2__leafrust | 3 |
| 1372-08-01 | F | *Sr22*_T_2__leafrust | 3 |
| 1372-08-01 | G | *Sr22*_T_2__leafrust | 3 |
| 1372-08-01 | H | *Sr22*_T_2__leafrust | 3 |
| Moore |  | Susceptible check | 3^+^ |
| PI584760 |  | Resistant check | 0; |
| Golden Promise |  | Wild type | 3 |

^†^Not assayed.

**Table S8** Puccinia hordei race 4 infection assays on Sr33 T_2_ homozygous lines.

| ID | Plant | Leaf number | Infection type |
| --- | --- | --- | --- |
| 1‒1 | 1 | 1 | 3 |
| 1‒2 | 1 | 2 | 3 |
| 1‒3 | 1 | 3 | 3 |
| 1‒4 | 1 | 4 | 3 |
| 1‒5 | 1 | 5 | 3 |
| 1‒6 | 1 | 6 | 33^+^ |
| 1‒7 | 1 | 7 | 3 |
| 1‒8 | 1 | 8 | 33^+^ |
| 1‒9 | 1 | 9 | 3 |
| 1‒10 | 1 | 10 | 3 |
| 1‒11 | 1 | 11 | 33^+^ |
| 1‒12 | 1 | 12 | 3 |
| 2‒1 | 2 | 1 | 33^+^ |
| 2‒2 | 2 | 2 | 33^+^ |
| 2‒3 | 2 | 3 | 33^+^ |
| 2‒4 | 2 | 4 | 3 |
| 2‒5 | 2 | 5 | 3 |
| 2‒6 | 2 | 6 | 3 |
| 2‒7 | 2 | 7 | 3 |
| 2‒8 | 2 | 8 | 33^+^ |
| 2‒9 | 2 | 9 | 3 |
| 2‒10 | 2 | 10 | 3 |
| 3‒1 | 3 | 1 | 3 |
| 3‒2 | 3 | 2 | 3 |
| 3‒3 | 3 | 3 | 3 |
| 3‒4 | 3 | 4 | 3 |
| 3‒5 | 3 | 5 | 3 |
| 3‒6 | 3 | 6 | 3 |
| 3‒7 | 3 | 7 | 3 |
| 3‒8 | 3 | 8 | 3 |
| 3‒9 | 3 | 9 | 3 |
| 3‒10 | 3 | 10 | 3 |
| 4‒1 | 4 | 1 | 3 |
| 4‒2 | 4 | 2 | 3 |
| 4‒3 | 4 | 3 | 3 |
| 4‒4 | 4 | 4 | 3 |
| 4‒5 | 4 | 5 | 3 |
| 4‒6 | 4 | 6 | 3 |
| 4‒7 | 4 | 7 | 3 |
| 4‒8 | 4 | 8 | 3 |
| 4‒9 | 4 | 9 | 3 |
| 4‒10 | 4 | 10 | 3 |
| Golden Promise |  | 1 | 33^+^ |
| Wheat *Sr33* |  | 1 | 0; |

**Table S9** Puccinia hordei race 4 infection assays on Sr33^d^ T_2_ families.

| Line ID | Plant ID | Line | Infection type |
| --- | --- | --- | --- |
| 1023-02-01 | D | *Sr33^d^*_T_2__leafrust | 2^+^3^-^ |
| 1023-02-01 | E | *Sr33^d^*_T_2__leafrust | 22^+^,3^-^ |
| 1023-02-01 | F | *Sr33^d^*_T_2__leafrust | 22^+^,3^-^ |
| 1023-02-01 | G | *Sr33^d^*_T_2__leafrust | 22^+^,3^-^ |
| 1023-06-01 | A | *Sr33^d^*_T_2__leafrust | 22^+^,3^-^ |
| 1023-06-01 | B | *Sr33^d^*_T_2__leafrust | 2^+^,3^-^ |
| 1023-06-01 | C | *Sr33^d^*_T_2__leafrust | NA^†^ |
| 1023-06-01 | D | *Sr33^d^*_T_2__leafrust | 22^+^ |
| 1023-06-01 | F | *Sr33^d^*_T_2__leafrust | NA |
| 1024-04-02 | A | *Sr33^d^*_T_2__leafrust | 2^+^3 |
| 1024-04-02 | B | *Sr33^d^*_T_2__leafrust | 2^+^3 |
| 1024-04-02 | C | *Sr33^d^*_T_2__leafrust | 2^+^,3^-^ |
| 1024-04-02 | E | *Sr33^d^*_T_2__leafrust | 22^+^ |
| 1024-06-01 | B | *Sr33^d^*_T_2__leafrust | NA |
| 1024-06-01 | D | *Sr33^d^*_T_2__leafrust | 23^-^,1^-^0; |
| 1024-06-01 | F | *Sr33^d^*_T_2__leafrust | 22^+^,3,1^+^ |
| 1024-06-01 | H | *Sr33^d^*_T_2__leafrust | 213^-^ |
| 1024-07-01 | B | *Sr33^d^*_T_2__leafrust | 213^-^ |
| 1024-07-01 | D | *Sr33^d^*_T_2__leafrust | 22^+^,13^-^ |
| 1024-07-01 | E | *Sr33^d^*_T_2__leafrust | 22^+^,3^-^ |
| 1024-07-01 | F | *Sr33^d^*_T_2__leafrust | 22^+^,3^-^ |
| 1024-11-01 | D | *Sr33^d^*_T_2__leafrust | 22^+^,3^-^ |
| 1024-11-01 | E | *Sr33^d^*_T_2__leafrust | 22^+^,3^-^ |
| 1024-11-01 | B | *Sr33^d^*_T_2__leafrust | 22^+^,3^-^1 |
| 1024-11-01 | A | *Sr33^d^*_T_2__leafrust | 22^+^,3^-^1 |
| 1024-13-01 | A | *Sr33^d^*_T_2__leafrust | 22^+^ |
| 1024-13-01 | B | *Sr33^d^*_T_2__leafrust | 22^+^1 |
| 1024-13-01 | C | *Sr33^d^*_T_2__leafrust | 2^+^3^-^ |
| 1024-13-01 | D | *Sr33^d^*_T_2__leafrust | 22^+^,3^-^ |
| 1024-13-01 | E | *Sr33^d^*_T_2__leafrust | NA |
| 1033-05-01 | A | *Sr33^d^*_T_2__leafrust | 22^+^,3^-^ |
| 1033-05-01 | B | *Sr33^d^*_T_2__leafrust | 22^+^,3^-^ |
| 1033-05-01 | C | *Sr33^d^*_T_2__leafrust | 22^+^,3^-^ |
| 1033-05-01 | G | *Sr33^d^*_T_2__leafrust | 22^+^,3^-^ |
| Moore |  | Susceptible check | 2^+^,3^-^ |
| PI584760 |  | Resistant check | 0;1^-^ |
| Golden Promise |  | Wild type | 3^-^,2^+^ |

^†^Not assayed.

**Table S10** Puccinia hordei race 4 infection assays on Sr35^d^ T_2_ families.

| Line ID | Plant ID | Line | Infection type |
| --- | --- | --- | --- |
| 1352-04-01 | A | *Sr35^d^*_T_2__leafrust | 3 |
| 1352-04-01 | B | *Sr35^d^*_T_2__leafrust | NA^†^ |
| 1352-04-01 | C | *Sr35^d^*_T_2__leafrust | NA |
| 1352-04-01 | D | *Sr35^d^*_T_2__leafrust | NA |
| 1352-04-01 | E | *Sr35^d^*_T_2__leafrust | 3^+^ |
| 1352-04-01 | F | *Sr35^d^*_T_2__leafrust | 3^+^ |
| 1352-04-01 | G | *Sr35^d^*_T_2__leafrust | 3^+^ |
| 1352-04-01 | H | *Sr35^d^*_T_2__leafrust | NA |
| 1352-04-02 | A | *Sr35^d^*_T_2__leafrust | 3 |
| 1352-04-02 | B | *Sr35^d^*_T_2__leafrust | 3^+^ |
| 1352-04-02 | C | *Sr35^d^*_T_2__leafrust | 3^+^ |
| 1352-04-02 | D | *Sr35^d^*_T_2__leafrust | NA |
| 1352-04-02 | E | *Sr35^d^*_T_2__leafrust | NA |
| 1352-04-02 | F | *Sr35^d^*_T_2__leafrust | 3 |
| 1352-04-02 | G | *Sr35^d^*_T_2__leafrust | 3 |
| 1352-04-02 | H | *Sr35^d^*_T_2__leafrust | NA |
| 1352-06-01 | A | *Sr35^d^*_T_2__leafrust | 3 |
| 1352-06-01 | B | *Sr35^d^*_T_2__leafrust | 3^-^,2 |
| 1352-06-01 | C | *Sr35^d^*_T_2__leafrust | 3 |
| 1352-06-01 | D | *Sr35^d^*_T_2__leafrust | NA |
| 1352-06-01 | E | *Sr35^d^*_T_2__leafrust | 3 |
| 1352-06-01 | F | *Sr35^d^*_T_2__leafrust | 3 |
| 1352-06-01 | G | *Sr35^d^*_T_2__leafrust | 3 |
| 1352-06-01 | H | *Sr35^d^*_T_2__leafrust | 3 |
| Moore |  | Susceptible check | 3^+^ |
| PI584760 |  | Resistant check | 0; |
| Golden Promise |  | Wild type | 3 |

^†^Not assayed.

**Table S11** Puccinia hordei race 4 infection assays on Sr45^d^ T_2_ families.

| Line ID | Plant ID | Line | Infection type |
| --- | --- | --- | --- |
| 1613-02-01 | A | *Sr45^d^*_T_2__leafrust | 3 |
| 1613-02-01 | D | *Sr45^d^*_T_2__leafrust | 3 |
| 1613-02-01 | E | *Sr45^d^*_T_2__leafrust | 3 |
| 1613-02-01 | G | *Sr45^d^*_T_2__leafrust | 3 |
| 1613-04-01 | A | *Sr45^d^*_T_2__leafrust | 3 |
| 1613-04-01 | E | *Sr45^d^*_T_2__leafrust | 2^+^3 |
| 1613-04-01 | G | *Sr45^d^*_T_2__leafrust | 33^+^ |
| 1613-05-01 | A | *Sr45^d^*_T_2__leafrust | 2^+^3 |
| 1613-05-01 | B | *Sr45^d^*_T_2__leafrust | 3 |
| 1613-05-01 | G | *Sr45^d^*_T_2__leafrust | 3 |
| 1613-05-01 | H | *Sr45^d^*_T_2__leafrust | 2^+^ |
| 1613-17-01 | B | *Sr45^d^*_T_2__leafrust | 33^+^ |
| 1613-17-01 | D | *Sr45^d^*_T_2__leafrust | 3 |
| 1613-17-01 | G | *Sr45^d^*_T_2__leafrust | 3^+^ |
| Moore |  | Susceptible check | 3^+^ |
| PI584760 |  | Resistant check | 0;1- |
| Golden Promise |  | Wild type | 33^+^ |

**Table S12** Stem rust infection assays with Pgt race MCCFC on Sr35^d^ T_1_ families.

| Plant ID | Line | Plant A | Plant B | Plant C | Plant D | Plant E | Plant F | Plant G | Plant H |
| --- | --- | --- | --- | --- | --- | --- | --- | --- | --- |
| 1350-01-01 | *Sr35^d^*_T_1_ | 2^+^3^-^ | 2^+^ | 2^+^3^-^ | 2^+^ | 2 | 2 | 2^+^3^-^ | 2^+^ |
| 1350-02-01 | *Sr35^d^*_T_1_ | 2^+^3^-^ | 2 | 2^+^3^-^ | 2^+^ | 2^+^3^-^ | 2^+^ | 2^+^ | 2 |
| 1352-02-01 | *Sr35^d^*_T_1_ | 2^+^3^-^ | 2 | 2^+^ | 2^+^3^-^ | 2^+^3^-^ | 2 | 2^+^ | 2^+^3^-^ |
| 1352-04-01 | *Sr35^d^*_T_1_ | 2 | NA | 2^+^3^-^ | 2^+^ | 2^+^ | 2^+^3^-^ | 2^+^ | 2 |
| 1352-04-02 | *Sr35^d^*_T_1_ | 2 | 2^+^ | 2^+^3^-^ | 2^+^ | 2 | 2^+^ | 2^+^3^-^ | 2 |
| 1352-04-03 | *Sr35^d^*_T_1_ | 2^+^3^-^ | 2^+^ | 2^+^3^-^ | 3 | 2^+^ | 2 | 2^+^ | 2 |
| 1352-06-01 | *Sr35^d^*_T_1_ | 2 | 2^+^ | 2^+^3^-^ | 1^+^ | 2^+^3^-^ | 2^+^3^-^ | 2^+^ | 2^+^ |
| 1352-07-01 | *Sr35^d^*_T_1_ | 2 | 2 | 3^+^ | 2^+^3^-^ | 2^+^ | 2^+^ | NA | 2^+^ |
| 1352-10-01 | *Sr35^d^*_T_1_ | 2 | 2^+^ | 1^-^ | 2 | 1 | 2^+^ | 2^+^3^-^ | NA |
| 1352-11-01 | *Sr35^d^*_T_1_ | 2^+^ | 2^+^3^-^ | 2^+^ | 2 | 2^+^ | 2^+^3^-^ | 2 | 2^+^ |
| GP-2015 | Golden Promise | 2^+^3^-^ | NA | NA | NA | NA | NA | NA | NA |
| GP- St Paul 2012 | Golden Promise | 2^+^3^-^ | NA | NA | NA | NA | NA | NA | NA |
| McNair | Susceptible control | 4 | 4 | NA | NA | NA | NA | NA | NA |

^†^Not assayed

**Table S13** Tiller number of *Hordeum vulgare* cv. Golden Promise with and without the presence of transgene.

|  |  |  |  |  |
| --- | --- | --- | --- | --- |
| **Gene** | **Line ID** | **Plant ID** | **Gene copy** | **Tiller number** |
| *Sr22* | 1370-11-01 | Plant 1 | 2 | 20 |
| *Sr22* | 1370-11-01 | Plant 3 | 2 | 23 |
| *Sr22* | 1370-11-01 | Plant 5 | 2 | 23 |
| *Sr22* | 1370-11-01 | Plant 8 | 2 | 21 |
| *Sr22* | 1370-11-01 | Plant 2 | 0 | 22 |
| *Sr22* | 1370-11-01 | Plant 15 | 0 | 23 |
| *Sr22* | 1370-11-01 | Plant 16 | 0 | 21 |
| *Sr33^d^* | 1023-06-01 | Plant 1 | 2 | 23 |
| *Sr33^d^* | 1023-06-01 | Plant 5 | 2 | 21 |
| *Sr33^d^* | 1024-13-01 | Plant 2 | 2 | 21 |
| *Sr33^d^* | 1024-13-01 | Plant 11 | 2 | 21 |
| *Sr33^d^* | 1024-13-01 | Plant 15 | 2 | 22 |
| *Sr33^d^* | 1023-06-01 | Plant 2 | 0 | 23 |
| *Sr33^d^* | 1023-06-01 | Plant 10 | 0 | 17 |
| *Sr33^d^* | 1024-13-01 | Plant 4 | 0 | 21 |
| *Sr33^d^* | 1024-13-01 | Plant 8 | 0 | 20 |
| *Sr33^d^* | 1024-13-01 | Plant 14 | 0 | 22 |
| *Sr35^d^* | 1352-04-01 | Plant 3 | 2 | 22 |
| *Sr35^d^* | 1352-04-01 | Plant 6 | 2 | 21 |
| *Sr35^d^* | 1352-04-01 | Plant 7 | 2 | 20 |
| *Sr35^d^* | 1352-04-02 | Plant 2 | 2 | 19 |
| *Sr35^d^* | 1352-04-02 | Plant 4 | 2 | 21 |
| *Sr35^d^* | 1352-04-02 | Plant 15 | 2 | 17 |
| *Sr35^d^* | 1352-04-01 | Plant 10 | 2 | 21 |
| *Sr35^d^* | 1352-06-01 | Plant 4 | 2 | 23 |
| *Sr35^d^* | 1352-04-01 | Plant 4 | 0 | 21 |
| *Sr35^d^* | 1352-04-01 | Plant 9 | 0 | 19 |
| *Sr35^d^* | 1352-04-01 | Plant 16 | 0 | 23 |
| *Sr35^d^* | 1352-04-02 | Plant 1 | 0 | 21 |
| *Sr35^d^* | 1352-04-02 | Plant 3 | 0 | 16 |
| *Sr35^d^* | 1352-04-02 | Plant 8 | 0 | 19 |
| *Sr35^d^* | 1352-06-01 | Plant 5 | 0 | 23 |
| *Sr35^d^* | 1352-06-01 | Plant 10 | 0 | 21 |
| *Sr35^d^* | 1352-06-01 | Plant 11 | 0 | 24 |
| *Sr45^d^* | 1613-04-01 | Plant 3 | 2 | 22 |
| *Sr45^d^* | 1613-04-01 | Plant 6 | 2 | 21 |
| *Sr45^d^* | 1613-05-01 | Plant 5 | 2 | 19 |
| *Sr45^d^* | 1613-05-01 | Plant 10 | 2 | 22 |
| *Sr45^d^* | 1613-17-01 | Plant 9 | 2 | 19 |
| *Sr45^d^* | 1613-17-01 | Plant 12 | 2 | 20 |
| *Sr45^d^* | 1613-17-01 | Plant 3 | 2 | 21 |
| *Sr45^d^* | 1613-04-01 | Plant 2 | 0 | 26 |
| *Sr45^d^* | 1613-04-01 | Plant 7 | 0 | 23 |
| *Sr45^d^* | 1613-05-01 | Plant 2 | 0 | 18 |
| *Sr45^d^* | 1613-05-01 | Plant 3 | 0 | 19 |
| *Sr45^d^* | 1613-05-01 | Plant 16 | 0 | 18 |
| *Sr45^d^* | 1613-17-01 | Plant 4 | 0 | 21 |
| *Sr45^d^* | 1613-17-01 | Plant 6 | 0 | 22 |
| *Sr45^d^* | 1613-17-01 | Plant 8 | 0 | 19 |
| *Sr45^d^* | 1613-17-01 | Plant 10 | 0 | 20 |

**Table S14** Thousand Grain Weight (TGW) of *Hordeum vulgare* cv. Golden Promise with and without the presence of transgene.

|  |  |  |  |  |
| --- | --- | --- | --- | --- |
| **Gene** | **Line ID** | **Plant ID** | **Gene copy** | **TGW (g)** |
| *Sr22* | 1370-11-01 | Plant 1 | 2 | 37.9 |
| *Sr22* | 1370-11-01 | Plant 3 | 2 | 40.9 |
| *Sr22* | 1370-11-01 | Plant 5 | 2 | 39.3 |
| *Sr22* | 1370-11-01 | Plant 8 | 2 | 41.8 |
| *Sr22* | 1370-11-01 | Plant 2 | 0 | 37.1 |
| *Sr22* | 1370-11-01 | Plant 15 | 0 | 43.0 |
| *Sr22* | 1370-11-01 | Plant 16 | 0 | 43.9 |
| *Sr33^d^* | 1023-06-01 | Plant 1 | 2 | 37.3 |
| *Sr33^d^* | 1023-06-01 | Plant 5 | 2 | 40.2 |
| *Sr33^d^* | 1023-06-01 | Plant 4 | 2 | 25.6 |
| *Sr33^d^* | 1024-13-01 | Plant 2 | 2 | 44.2 |
| *Sr33^d^* | 1024-13-01 | Plant 11 | 2 | 44.2 |
| *Sr33^d^* | 1024-13-01 | Plant 15 | 2 | 41.8 |
| *Sr33^d^* | 1023-06-01 | Plant 2 | 0 | 37.5 |
| *Sr33^d^* | 1023-06-01 | Plant 10 | 0 | 34.4 |
| *Sr33^d^* | 1023-06-01 | Plant 9 | 0 | 26.8 |
| *Sr33^d^* | 1024-13-01 | Plant 4 | 0 | 40.4 |
| *Sr33^d^* | 1024-13-01 | Plant 8 | 0 | 41.5 |
| *Sr33^d^* | 1024-13-01 | Plant 14 | 0 | 37.5 |
| *Sr35^d^* | 1352-04-01 | Plant 3 | 2 | 35.2 |
| *Sr35^d^* | 1352-04-01 | Plant 6 | 2 | 33.9 |
| *Sr35^d^* | 1352-04-01 | Plant 7 | 2 | 32.9 |
| *Sr35^d^* | 1352-04-01 | Plant 10 | 2 | 37.9 |
| *Sr35^d^* | 1352-04-02 | Plant 2 | 2 | 27.5 |
| *Sr35^d^* | 1352-04-02 | Plant 4 | 2 | 26.5 |
| *Sr35^d^* | 1352-04-02 | Plant 15 | 2 | 26.3 |
| *Sr35^d^* | 1352-06-01 | Plant 4 | 2 | 39.5 |
| *Sr35^d^* | 1352-04-01 | Plant 4 | 0 | 39.4 |
| *Sr35^d^* | 1352-04-01 | Plant 9 | 0 | 35.3 |
| *Sr35^d^* | 1352-04-01 | Plant 16 | 0 | 39.6 |
| *Sr35^d^* | 1352-04-02 | Plant 1 | 0 | 33.0 |
| *Sr35^d^* | 1352-04-02 | Plant 3 | 0 | 30.6 |
| *Sr35^d^* | 1352-04-02 | Plant 8 | 0 | 31.1 |
| *Sr35^d^* | 1352-06-01 | Plant 5 | 0 | 36.3 |
| *Sr35^d^* | 1352-06-01 | Plant 10 | 0 | 40.3 |
| *Sr35^d^* | 1352-06-01 | Plant 11 | 0 | 39.3 |
| *Sr45^d^* | 1613-04-01 | Plant 3 | 2 | 42.5 |
| *Sr45^d^* | 1613-04-01 | Plant 6 | 2 | 43.1 |
| *Sr45^d^* | 1613-05-01 | Plant 5 | 2 | 42.8 |
| *Sr45^d^* | 1613-05-01 | Plant 10 | 2 | 39.5 |
| *Sr45^d^* | 1613-05-01 | Plant 4 | 2 | 44.1 |
| *Sr45^d^* | 1613-17-01 | Plant 9 | 2 | 37.4 |
| *Sr45^d^* | 1613-17-01 | Plant 12 | 2 | 36.4 |
| *Sr45^d^* | 1613-17-01 | Plant 3 | 2 | 43.1 |
| *Sr45^d^* | 1613-04-01 | Plant 2 | 0 | 42.8 |
| *Sr45^d^* | 1613-04-01 | Plant 7 | 0 | 43.0 |
| *Sr45^d^* | 1613-05-01 | Plant 2 | 0 | 38.2 |
| *Sr45^d^* | 1613-05-01 | Plant 3 | 0 | 37.3 |
| *Sr45^d^* | 1613-05-01 | Plant 16 | 0 | 33.0 |
| *Sr45^d^* | 1613-17-01 | Plant 4 | 0 | 35.5 |
| *Sr45^d^* | 1613-17-01 | Plant 6 | 0 | 39.0 |
| *Sr45^d^* | 1613-17-01 | Plant 8 | 0 | 40.3 |
| *Sr45^d^* | 1613-17-01 | Plant 10 | 0 | 37.8 |

**Table S15** Development stages of *Sr22* transgenic under speed breeding condition. Values indicated are expressed as days after sowing (DAS).

|  |  |  | **Development stage**^†^ | | | | | |
| --- | --- | --- | --- | --- | --- | --- | --- | --- |
| **Line ID** | **Plant ID** | **Gene copy** | **3-leaf stage** | **GS33** | **GS50** | **GS57** | **GS65** | **GS85** |
| 1370-11-01 | Plant 1 | 2 | 15 | 25 | NA^‡^ | 50 | 45 | 53 |
| 1370-11-01 | Plant 3 | 2 | 15 | 27 | 39 | 50 | 45 | 53 |
| 1370-11-01 | Plant 5 | 2 | 15 | 27 | 39 | 50 | NA | 53 |
| 1370-11-01 | Plant 8 | 2 | 15 | 27 | 41 | 50 | 47 | 62 |
| 1370-11-01 | Plant 2 | 0 | 15 | 27 | 41 | 47 | 47 | 58 |
| 1370-11-01 | Plant 15 | 0 | 15 | 26 | NA | 47 | 45 | 53 |
| 1370-11-01 | Plant 16 | 0 | 15 | 27 | 41 | 50 | 47 | 53 |

^†^Growth stages measured for the first tiller according to the Zadoks’ Scale (Zadoks et al., 1974).

^‡^Not available.

**Table S16** Development stages of *Sr33^d^* transgenic under speed breeding conditions. Values indicated are expressed as days after sowing (DAS).

|  |  |  | **Development stage**^†^ | | | | | |
| --- | --- | --- | --- | --- | --- | --- | --- | --- |
| **Line ID** | **Plant ID** | **Gene copy** | **3-leaf stage** | **GS33** | **GS50** | **GS57** | **GS65** | **GS85** |
| 1023-06-01 | Plant 1 | 2 | 15 | 27 | 39 | NA^‡^ | 47 | 58 |
| 1023-06-01 | Plant 4 | 2 | 15 | 27 | 41 | 45 | 47 | 58 |
| 1023-06-01 | Plant 5 | 2 | 15 | 27 | 47 | 58 | NA | NA |
| 1024-13-01 | Plant 2 | 2 | 15 | 27 | 41 | 50 | NA | 53 |
| 1024-13-01 | Plant 15 | 2 | 15 | 25 | NA | 45 | 45 | 58 |
| 1024-13-01 | Plant 11 | 2 | 15 | 27 | NA | NA | 50 | NA |
| 1023-06-01 | Plant 2 | 0 | 15 | 25 | NA | 45 | 45 | 53 |
| 1023-06-01 | Plant 9 | 0 | 15 | 27 | 41 | 62 | 58 | NA |
| 1023-06-01 | Plant 10 | 0 | 15 | 25 | 47 | 62 | 50 | 62 |
| 1023-06-01 | Plant 11 | 0 | 15 | 25 | 39 | 47 | 45 | NA |
| 1024-13-01 | Plant 4 | 0 | 15 | 25 | 39 | 47 | 45 | 58 |
| 1024-13-01 | Plant 8 | 0 | 15 | 25 | 39 | NA | 45 | 58 |
| 1024-13-01 | Plant 14 | 0 | 15 | 25 | NA | 41 | 45 | 53 |

^†^Growth stages measured for the first tiller according to the Zadoks’ Scale (Zadoks et al., 1974).

^‡^Not available.

**Table S17** Development stages of *Sr35^d^* transgenic under speed breeding conditions. Values indicated are expressed as days after sowing (DAS).

|  |  |  | **Development stage**^†^ | | | | | |
| --- | --- | --- | --- | --- | --- | --- | --- | --- |
| **Line ID** | **Plant ID** | **Gene copy** | **3-leaf stage** | **GS33** | **GS50** | **GS57** | **GS65** | **GS85** |
| 1352-04-01 | Plant 3 | 2 | 15 | NA^‡^ | 39 | 45 | 45 | 50 |
| 1352-04-01 | Plant 6 | 2 | 15 | 26 | 39 | 45 | 45 | 58 |
| 1352-04-01 | Plant 7 | 2 | 15 | 26 | NA | 45 | 45 | 53 |
| 1352-04-01 | Plant 10 | 2 | 15 | 27 | 41 | 50 | 45 | 53 |
| 1352-04-02 | Plant 2 | 2 | 15 | 25 | 41 | 47 | 45 | 58 |
| 1352-04-02 | Plant 4 | 2 | 15 | 27 | NA | 47 | 45 | 58 |
| 1352-04-02 | Plant 15 | 2 | 15 | 26 | 41 | 47 | NA | 62 |
| 1352-06-01 | Plant 4 | 2 | 15 | 25 | NA | 47 | NA | 53 |
| 1352-04-01 | Plant 4 | 0 | 15 | 26 | 39 | 46 | 45 | 53 |
| 1352-04-01 | Plant 9 | 0 | 15 | 25 | 50 | 58 | NA | NA |
| 1352-04-01 | Plant 16 | 0 | 15 | 25 | 39 | 50 | 45 | 53 |
| 1352-04-02 | Plant 1 | 0 | 15 | NA | NA | 45 | 45 | 53 |
| 1352-04-02 | Plant 3 | 0 | 15 | 26 | 41 | 47 | NA | NA |
| 1352-04-02 | Plant 8 | 0 | 15 | 26 | NA | NA | 45 | 53 |
| 1352-04-02 | Plant 16 | 0 | 15 | 27 | 58 | NA | NA | NA |
| 1352-06-01 | Plant 5 | 0 | 15 | 25 | NA | 45 | NA | 58 |
| 1352-06-01 | Plant 10 | 0 | 15 | 25 | NA | 47 | NA | 58 |
| 1352-06-01 | Plant 11 | 0 | 15 | 25 | NA | 41 | NA | 62 |

^†^Growth stages measured for the first tiller according to the Zadoks’ Scale (Zadoks et al., 1974).

^‡^Not available.

**Table S18** Development stages of *Sr45^d^* transgenic under speed breeding conditions. Values indicated are expressed as days after sowing (DAS).

|  |  |  | **Development stage**^†^ | | | | | |
| --- | --- | --- | --- | --- | --- | --- | --- | --- |
| **Line ID** | **Plant ID** | **Gene copy** | **3-leaf stage** | **GS33** | **GS50** | **GS57** | **GS65** | **GS85** |
| 1613-04-01 | Plant 3 | 2 | 15 | 26 | 39 | 58 | 53 | 58 |
| 1613-04-01 | Plant 6 | 2 | 15 | 27 | NA^‡^ | 45 | 45 | 58 |
| 1613-17-01 | Plant 3 | 2 | 15 | 25 | NA | 45 | 45 | 58 |
| 1613-17-01 | Plant 9 | 2 | 15 | 25 | 41 | 58 | 50 | NA |
| 1613-17-01 | Plant 12 | 2 | 15 | 25 | 39 | 45 | 45 | 53 |
| 1613-05-01 | Plant 4 | 2 | 15 | 26 | NA | 58 | 45 | NA |
| 1613-05-01 | Plant 5 | 2 | 15 | 25 | 41 | NA | NA | 58 |
| 1613-05-01 | Plant 10 | 2 | 15 | 27 | 41 | 45 | 47 | 53 |
| 1613-04-01 | Plant 2 | 0 | 15 | NA | NA | 50 | 53 | 58 |
| 1613-04-01 | Plant 7 | 0 | 15 | 27 | NA | 53 | 47 | 58 |
| 1613-17-01 | Plant 4 | 0 | 15 | 25 | NA | 41 | NA | 53 |
| 1613-17-01 | Plant 6 | 0 | 15 | 25 | 39 | 47 | 45 | 62 |
| 1613-17-01 | Plant 8 | 0 | 15 | 25 | NA | 41 | 45 | 53 |
| 1613-17-01 | Plant 10 | 0 | 15 | 25 | 39 | 47 | NA | 62 |
| 1613-05-01 | Plant 2 | 0 | 15 | NA | NA | 50 | 53 | 58 |
| 1613-05-01 | Plant 3 | 0 | 15 | 27 | 41 | 47 | 47 | 53 |
| 1613-05-01 | Plant 16 | 0 | 15 | 27 | 41 | 47 | 47 | 62 |

^†^Growth stages measured for the first tiller according to the Zadoks’ Scale (Zadoks et al., 1974).

^‡^Not available.

**Table S19** Functional testing of *Sr33* and *Sr45* with *Pgt* race MCCFC. The infection types were scored according to the Stakman infection type scale (Stakman et al. 1962), and the scores ranged from: resistant (0;, 1-, 1), moderately resistant (1+, 2), moderately susceptible (2+, 3-, 3), to susceptible (3+, 4).

| **Gene postulation** | **Accession** | **MCCFC score** |
| --- | --- | --- |
| Only *Sr33* present | TOWWC153 | 1- |
| Only *Sr45* present | TOWWC070 | 0; |
| Only *Sr45* present | TOWWC084 | 0; |
| Only *Sr45* present | TOWWC139 | 0; |
| Only *Sr45* present | TOWWC191 | 0; |
| *Sr33, Sr45, Sr46, SrTA1662* absent | TOWWC009 | 3- |
| *Sr33, Sr45, Sr46, SrTA1662* absent | TOWWC013 | 2+ |
| *Sr33, Sr45, Sr46, SrTA1662* absent | TOWWC026 | 2 |
| *Sr33, Sr45, Sr46, SrTA1662* absent | TOWWC027 | 2+ |
| *Sr33, Sr45, Sr46, SrTA1662* absent | TOWWC031 | 2+ |
| *Sr33, Sr45, Sr46, SrTA1662* absent | TOWWC045 | 2+ |
| *Sr33, Sr45, Sr46, SrTA1662* absent | TOWWC056 | 2+ |
| *Sr33, Sr45, Sr46, SrTA1662* absent | TOWWC057 | 3 |
| *Sr33, Sr45, Sr46, SrTA1662* absent | TOWWC058 | 3 |
| *Sr33, Sr45, Sr46, SrTA1662* absent | TOWWC059 | 3 |
| *Sr33, Sr45, Sr46, SrTA1662* absent | TOWWC063 | 3+ |
| *Sr33, Sr45, Sr46, SrTA1662* absent | TOWWC069 | 2+ |
| *Sr33, Sr45, Sr46, SrTA1662* absent | TOWWC071 | 3 |
| *Sr33, Sr45, Sr46, SrTA1662* absent | TOWWC092 | 3- |
| *Sr33, Sr45, Sr46, SrTA1662* absent | TOWWC095 | 3- |
| *Sr33, Sr45, Sr46, SrTA1662* absent | TOWWC100^†^ | 1- |
| *Sr33, Sr45, Sr46, SrTA1662* absent | TOWWC127 | 2+ |
| *Sr33, Sr45, Sr46, SrTA1662* absent | TOWWC133 | 2 |
| *Sr33, Sr45, Sr46, SrTA1662* absent | TOWWC137 | 2 |
| *Sr33, Sr45, Sr46, SrTA1662* absent | TOWWC148 | 3- |
| *Sr33, Sr45, Sr46, SrTA1662* absent | TOWWC149 | 2+ |
| *Sr33, Sr45, Sr46, SrTA1662* absent | TOWWC162 | 2- |
| *Sr33, Sr45, Sr46, SrTA1662* absent | TOWWC163 | 3- |
| *Sr33, Sr45, Sr46, SrTA1662* absent | TOWWC164 | 2+ |
| *Sr33, Sr45, Sr46, SrTA1662* absent | TOWWC171 | 3 |
| *Sr33, Sr45, Sr46, SrTA1662* absent | TOWWC176 | 3+ |
| *Sr33, Sr45, Sr46, SrTA1662* absent | TOWWC177 | 3+ |
| *Sr33, Sr45, Sr46, SrTA1662* absent | TOWWC178 | 3+ |
| *Sr33, Sr45, Sr46, SrTA1662* absent | TOWWC179 | 3 |
| *Sr33, Sr45, Sr46, SrTA1662* absent | TOWWC187 | 3- |
| *Sr33, Sr45, Sr46, SrTA1662* absent | TOWWC193 | 3 |

^†^The resistance observed in TOWWC100 might be caused by a version of *Sr33*, *Sr45*, *Sr46* or *SrTA1662* which escaped our sequence-homology cut-off, or by the presence of an altogether novel *Sr* gene.

**Table S20** Photosynthetic photon flux density (PPFD) measurements for the LED-supplemented glasshouse. PPFD was measured in μmol m^-2^ s^-1^ at a central location using an UPRTek MK350S spectrophotometer and associated uSpectrum software (UPRTek, Taiwan). Values are the mean of eighteen measurements ± the standard deviation taken in a metre square area under a light fixture. Plants were rotated around on a weekly basis.

| **Position** | **Night** |
| --- | --- |
| Bench height^†^ | 303.1 ± 67.87 |
| Canopy height^‡^ | 467.0 ± 132.55 |

^†^Bench height was 75 cm from the light fixture.

^‡^Canopy height was 18 cm from the light fixture.
